# Conceptualizing pathways linking women’s empowerment and prematurity in developing countries

**DOI:** 10.1186/s12884-017-1502-6

**Published:** 2017-11-08

**Authors:** Patience A. Afulani, Molly Altman, Joseph Musana, May Sudhinaraset

**Affiliations:** 1 Preterm Birth Initiative, University of California, San Francisco (UCSF), San Francisco, CA USA; 2 UCSF School of Medicine, San Francisco, CA USA; 3 UCSF School of Nursing, San Francisco, CA USA

**Keywords:** Women, Empowerment, Autonomy, Prematurity, Preterm, Model, Framework, Stress, Developing countries

## Abstract

**Background:**

Globally, prematurity is the leading cause of death in children under the age of 5. Many efforts have focused on clinical approaches to improve the survival of premature babies. There is a need, however, to explore psychosocial, sociocultural, economic, and other factors as potential mechanisms to reduce the burden of prematurity. Women’s empowerment may be a catalyst for moving the needle in this direction. The goal of this paper is to examine links between women’s empowerment and prematurity in developing settings. We propose a conceptual model that shows pathways by which women’s empowerment can affect prematurity and review and summarize the literature supporting the relationships we posit. We also suggest future directions for research on women’s empowerment and prematurity.

**Methods:**

The key words we used for empowerment in the search were “empowerment,” “women’s status,” “autonomy,” and “decision-making,” and for prematurity we used “preterm,” “premature,” and “prematurity.” We did not use date, language, and regional restrictions. The search was done in PubMed, Population Information Online (POPLINE), and Web of Science. We selected intervening factors—factors that could potentially mediate the relationship between empowerment and prematurity—based on reviews of the risk factors and interventions to address prematurity and the determinants of those factors.

**Results:**

There is limited evidence supporting a direct link between women’s empowerment and prematurity. However, there is evidence linking several dimensions of empowerment to factors known to be associated with prematurity and outcomes for premature babies. Our review of the literature shows that women’s empowerment may reduce prematurity by (1) preventing early marriage and promoting family planning, which will delay age at first pregnancy and increase interpregnancy intervals; (2) improving women’s nutritional status; (3) reducing domestic violence and other stressors to improve psychological health; and (4) improving access to and receipt of recommended health services during pregnancy and delivery to help prevent prematurity and improve survival of premature babies.

**Conclusions:**

Women’s empowerment is an important distal factor that affects prematurity through several intervening factors. Improving women’s empowerment will help prevent prematurity and improve survival of preterm babies. Research to empirically show the links between women’s empowerment and prematurity is however needed.

**Electronic supplementary material:**

The online version of this article (doi:10.1186/s12884-017-1502-6) contains supplementary material, which is available to authorized users.

## Background

Every year, approximately 15 million babies are born preterm—equating to greater than 1 in 10 babies [1, 2]. Preterm birth is defined as birth before 37 completed weeks of gestation [2]. Preterm birth is a global issue, affecting about 12% of births in low-income countries and about 9% of births in high-income countries [2]. More than 60% of preterm births, however, occur in sub-Saharan Africa (SSA) and South Asia [2]. Evidence from countries with reliable trend data suggest preterm birth rates are increasing [3, 4]. Data from low- and middle-income countries also suggest increasing preterm birth rates in some countries, although changes in the types of data collected and the measurement of gestational age in these countries limit trend analysis [3].

Globally, prematurity is the leading cause of death in children under the age of 5, with more than 1 million children dying from direct complications of preterm birth [5, 6]. Prematurity also increases a baby’s risk of dying from other causes, especially from neonatal infections, with preterm birth estimated to be a risk factor in at least 50% of all neonatal deaths [7, 8]. However, the risk of a neonatal death due to complications of prematurity is much higher in developing settings: a baby born prematurely in Africa is about 12 times more likely to die than a baby born prematurely in Europe. SSA and South Asia account for more than 80% of the world’s 1.1 million deaths due to prematurity [1, 9, 10].

In addition to the significant contribution to mortality, preterm birth is a major source of morbidity, with many preterm babies who survive facing a lifetime of disability. Sequelae from preterm birth include impaired neuro-developmental functioning from increased risk of cerebral palsy, learning impairment, visual, hearing, and psychomotor problems, as well as a higher risk of non-communicable disease such as hypertension and asthma [1, 2, 11–13]. The effects of preterm birth exert a heavy burden on families, society, and the health system [2].

Many efforts have focused on improving the survival of infants born prematurely, and indeed, more than three-quarters of premature infants can be saved with feasible, cost-effective care during childbirth [1, 14, 15]. Yet in developing countries about half of babies are born at home without the support of skilled birth attendants. Even for those born in a health facility, essential newborn care is often lacking. The low skilled attendance accounts for the huge disparity in the survival of premature babies in developed and developing settings [1, 9, 10]. The factors accounting for the disparity in preterm birth rates, as well as the low skilled attendance, however, extend beyond clinical interventions.

There is thus a need to further explore psychosocial, sociocultural, economic, political, and legal factors as potential mechanisms that may reduce the overall prevalence of preterm birth and improve outcomes for premature babies in developing settings. Women’s empowerment may be a catalyst for examining these factors to facilitate efforts towards reducing the global burden of prematurity. While women’s empowerment is recognized as one of the strategies to reduce the burden of prematurity [1, 16, 17], women’s empowerment in relation to prematurity seems to be referenced with caution. In “Born Too Soon: The Global Action Report on Preterm Birth,” empowerment is mentioned only once (three times if you count the repetition in the summary and diagram) in the 124-page report—only in relation to family planning as part of preconception care [1]. This seems to suggest the only recognized link between empowerment and prematurity is family planning. However, we believe that there is a broader relationship between women’s empowerment and prematurity.

Our goal in this paper is to illustrate the links between women’s empowerment and prematurity in developing settings. We propose a conceptual model that shows pathways by which women’s empowerment can affect prematurity, drawing on the literature on factors associated with prematurity and how these factors are related to women’s empowerment. Explicating how women’s empowerment might affect prematurity will help stimulate research on the topic and help promote women’s empowerment as a viable strategy to prevent prematurity. We also suggest potential research questions to provide direction for future research on the role of women’s empowerment in preventing prematurity and improving the survival of premature babies.

### Risk factors for prematurity

Preterm birth is a syndrome with a variety of causes and underlying factors, which can be classified into spontaneous preterm births and provider-initiated preterm births [2, 6, 18, 19]. Spontaneous preterm births occur from spontaneous onset of labor or following prelabor premature rupture of membranes (PPROM) before 37 completed weeks of gestation. Spontaneous preterm birth is a multifactorial process, resulting from the interplay of genetic, social, and environmental factors among others. The cause of spontaneous preterm labor, however, remains unidentified in about a third to up to half of all cases [20–22]. Numerous risk factors have been identified for spontaneous preterm birth, falling into several categories, as shown in Table 1 [1, 2].

**Table 1:** Risk factors for spontaneous preterm birth and examples of interventions

Risk factor	Examples	Interventions
Age at pregnancy and pregnancy spacing	Adolescent pregnancy, advanced maternal age, or short interpregnancy interval	Preconception care, including encouraging family planning beginning in adolescence and continuing between pregnancies
Nutrition	Undernutrition, obesity, micronutrient deficiencies	Improve nutritional status prior to conception and throughout pregnancy
Maternal psychological health	Depression, violence against women	Behavioral and community interventions to prevent violence against women
Multiple pregnancy	Increased rates of twin and higher order pregnancies with assisted reproduction	Introduction and monitoring of policies for best practice in assisted reproduction
Infection	Urinary tract infections, malaria, HIV, syphilis, bacterial vaginosis	Sexual health programs aimed at prevention and treatment of infections prior to pregnancy. Specific interventions to prevent infections and mechanisms for early detection and treatment of infections occurring during pregnancy
Underlying maternal chronic medical conditions	Diabetes, hypertension, anemia, asthma, thyroid disease	Improve control prior to conception and throughout pregnancy
Lifestyle/work related	Smoking, excess alcohol consumption, recreational drug use, excess physical work/activity	Behavior and community interventions targeting all women of childbearing age in general and pregnant women in particular through antenatal care with early detection and treatment of pregnancy complications
Genetic and other	Genetic risk, e.g., family history of cervical incompetence	Identification and management of pregnant women at higher risk of preterm birth

Source: Adapted from “Born Too Soon: The Global Action Report on Preterm Birth” [1]

Three of the known risk factors for spontaneous preterm birth are interrelated sociodemographic factors related to timing of pregnancies: maternal age, interpregnancy intervals, and parity. The risk of preterm birth is higher with young or advanced maternal age, short interpregnancy intervals, and high parity [22]. For example, one study found that after adjusting for confounders, maternal age less than 17 years was associated with almost two times higher odds of preterm birth compared to maternal age 20 to 24 years [23]. In another study, an interpregnancy interval of less than 6 months increased the risk of preterm birth by more than two times when compared to birth intervals of 18–23 months [24]. One potential explanation for these findings is that adolescents’ bodies are not physically prepared for pregnancy and childbirth, and they do not have the nutritional reserves to maintain a pregnancy to term [1]. For interpregnancy interval and parity, maternal depletion is thought to be an underlying factor, as pregnancy consumes maternal stores of essential vitamins, minerals, and amino acids, and rapid succession of pregnancies decreases the opportunity to replenish these nutrients [22]. Adolescent pregnancies also tend to be associated with other risk factors (which we discuss below) such as increased risk of sexually transmitted infection, domestic violence, and lack of access to health care [1].

Another set of risk factors relates to maternal nutrition. Poor nutritional status based on different types of indicators is associated with higher risk of preterm birth [22]. For example, one study found that women with a body mass index (BMI) of less than 19 kg/m^2^ had significantly higher preterm birth rates than women with higher BMIs (a spontaneous preterm birth rate of 17% compared to 8% for those with a BMI of 25–29.9 kg/m^2^) [25]. Women with low serum concentrations of iron, folate, and zinc are also more likely to have preterm births than those with normal concentrations of these nutrients [22, 26, 27]. Potential mechanisms for the effect of nutritional status on preterm birth include decreased blood volume and reduced uterine blood flow, increased maternal infections and chronic conditions from lack of essential vitamins and minerals, and increased risk of congenital anomalies such as neural tube defects, which are associated with increased risk of prematurity [22].

In addition, there is strong evidence that maternal psychological health, including stress, depression, pregnancy-related anxiety, and violence against women, contribute significantly to increased risk of spontaneous preterm birth [1, 28–35]. Several studies have shown that women who experience high levels of stress, including acute and chronic stress, as well as psychological or social stress such as housing instability and severe material hardship, are almost two times more likely to have a preterm birth [22, 34, 36]. Even adverse childhood experiences, which are a source of stress during childhood, have been linked to increased risk of prematurity later in life [37]. Women who experience domestic violence, a major source of stress among women in developing settings, are also about two times more likely to have preterm births [1, 31, 38]. Stress is thought to contribute to preterm birth through both direct physiological mechanisms, such as increased inflammation, as well as behavioral pathways like smoking, drug, and alcohol abuse [22, 39].

Other risk factors for spontaneous preterm birth include pregnancy risk factors such as multiple pregnancies, intrauterine growth restriction, and congenital abnormalities from various causes; maternal infections and medical conditions; behavioral factors such as smoking; and those thought to be indicative of genetic risks, including maternal history of preterm birth [2, 21, 22, 40, 41]. There are also more complex factors such as socioeconomic status and race/ethnicity, whose effects are likely mediated by other risk factors [1, 29, 30, 42–44].

The causes of provider-initiated preterm birth (i.e., induction of labor or elective caesarean birth before 37 completed weeks of gestation for maternal or fetal indications) are also numerous [2, 19]. Clinical conditions increasing the risk of provider-initiated preterm birth include severe pre-eclampsia and eclampsia, placental abruption, uterine rupture, and fetal distress [45]. Some provider-initiated preterm births, however, have no strong medical indication and are sometimes due to errors in gestational age assessment or other non-medical factors [46, 47]. In addition, both maternal and fetal factors are more frequently seen in pregnancies occurring after assisted fertility treatments, thus increasing the risk of both spontaneous and provider-initiated preterm births for these women [47, 48]. However, the contribution to preterm births from assisted fertility treatments is relatively small in developing countries.

### Recommended interventions to address the burden of prematurity

Addressing the burden of prematurity is two-faceted, involving (1) prevention of prematurity and (2) care of the premature baby. Interventions with proven effect for prematurity prevention are clustered in the preconception period, between pregnancy and pregnancy periods, as well as during preterm labor, while interventions to reduce death and disability among premature babies are clustered during labor and after birth [1, 17]. The interventions thus fall along a continuum of care starting before conception and continuing through pregnancy to after delivery [1, 49]. It is estimated that if interventions with proven benefit for preterm birth prevention and reduction of neonatal complications were universally available to women and their babies (i.e., 95% coverage), almost one million premature babies could be saved each year [1].

In the preconception period, family planning is a highly cost-effective approach, especially in regions with high rates of adolescent pregnancy, as it addresses some of the major risk factors for preterm birth—timing of first pregnancy, short interpregnancy intervals, and high parity [1, 16]. Other preconception interventions are shown in Table 1. Antenatal care (ANC) is a service delivery system through which women can be reached at multiple times during pregnancy with a package of interventions that can prevent prematurity or improve outcomes for premature babies. Basic services that can be delivered during ANC with potential impact on reducing preterm birth rates include screening for and treatment of infections, including sexually transmitted diseases, counseling on birth preparation and warning symptoms for identification of early labor and other risk factors, and other services listed in Table 1 [1, 17].

Good quality care during labor and delivery can improve outcomes for premature babies. Lack of access to high quality care at delivery is, however, a major problem in developing settings [1, 17].

### Potential intervening factors for a relationship between women’s empowerment and prematurity

Some of the risk factors and interventions described above are likely factors that mediate a relationship between women’s empowerment and prematurity. We hypothesize that increasing women’s empowerment will reduce the burden of prematurity by reducing incidence of some risk factors for prematurity and promoting health-seeking behaviors that can prevent prematurity and improve the survival of premature babies. In this paper we focus on four of these intervening factors, drawn from our review of the risk factors and interventions to address prematurity. These are:

1. Delayed first pregnancy and increased interpregnancy intervals
2. Improved nutrition
3. Improved psychological health
4. Receipt of good quality maternal health care

These four factors are especially important in developing settings where adolescent pregnancies and short interpregnancy intervals, coupled with poor nutrition, are major issues; where many women are exposed to stressors such as gender-based violence; and where access to good quality health care is a lingering problem. Given the burden of these problems in developing settings, addressing them has great potential for preventing prematurity and improving the survival of premature babies [1].

**Fig. 1:**
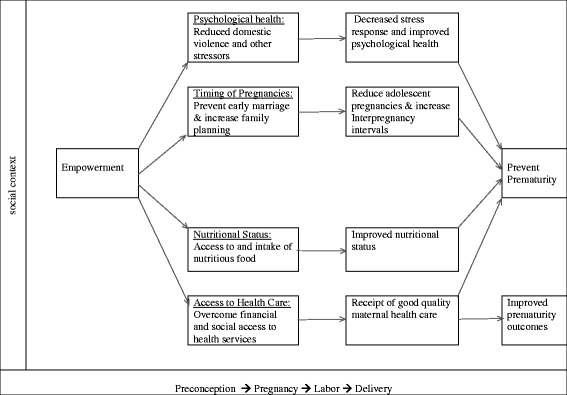
Pathways linking women’s empowerment and prematurity

While there are other potential intervening factors, we select these four factors to illustrate examples of pathways by which women’s empowerment may affect prematurity. These four intervening factors were selected based on our assessment of which of the risk factors or recommended interventions for prematurity had plausible links to women’s empowerment. We intentionally avoided proposing linkages between empowerment and the biological risk factors for prematurity because of the complexity of trying to validate such relationships. However, we believe plausible linkages exist with these four factors, because they have many social determinants. These factors are meant to be illustrative and not exhaustive, and they could be expanded to include others. By proposing a conceptual framework around women’s empowerment and prematurity, we aim to provide future direction for new and innovative interventions to address prematurity that include dimensions of women’s empowerment. The relationships we posit are shown in Fig. 1, and we summarize the literature supporting these relationships in the results.

## Methods

To provide evidence for how empowerment may be related to prematurity, we first searched the literature using key words related to empowerment and prematurity. The key words we used for empowerment were “empowerment,” “women’s status,” “autonomy,” and “decision-making,” and for prematurity we used “preterm,” “premature,” and “prematurity.” The only search restriction was restriction to human studies. We did not use date, language, and regional restrictions. The search was done in PubMed, Population Information Online (POPLINE), and Web of Science. We also did a quick search in Google for the gray literature, although this search was not exhaustive. Based on our initial scoping review of the literature on this topic, there were not sufficient relevant articles on empowerment and prematurity to warrant a systematic review.

Next we examined the literature on the risk factors for prematurity and the recommended interventions to prevent prematurity or improve the survival of premature babies (summarized above). We then identified factors from this review that have a plausible link with empowerment and thus could be potential intervening factors between women’s empowerment and prematurity. This process was guided by our prior knowledge as well as a scoping review of literature on the determinants of the risk factors and interventions. We then reviewed and summarized the literature supporting any links between empowerment and these factors. We used the same key words for empowerment and added various terms for the intervening factors, such as “age at first pregnancy,” “interpregnancy interval,” “maternal nutrition,” “stress,” “antenatal care,” “skilled birth attendants,” etc.

We considered the different but interrelated dimensions of empowerment, as well as the different ways of measuring empowerment seen in the extant literature [50–56]. While we recognize the complexity of measuring empowerment, we did not try to disentangle issues of measurement in this paper. However, we tried to evaluate which dimensions of empowerment were captured by the measures of empowerment used in various studies. Using the available literature, we then constructed a conceptual framework with the intervening factors to connect women’s empowerment and prematurity (Fig. 1). The focus of this framework is on developing countries; thus, we first looked specifically for literature from developing countries on the intervening factors. We, however, also drew on literature from developed countries to supplement those areas where less work is available in developing settings to provide evidence on suggested pathways [57–59]. The reviews were not intended to be systematic reviews; hence, the literature discussed is intended to be representative rather than comprehensive.

### Conceptualizing women’s empowerment

Women’s empowerment is a complex construct and has been extensively discussed [50–53, 55, 60]. In this paper we use Kabeer’s definition of women’s empowerment: “The expansion of people’s ability to make strategic life choices in a context where this ability was previously denied to them” [50]. Empowerment in this sense incorporates three interrelated components: resources (including access and future claims to material, human, and social resources); agency (including processes of decision-making, as well as less measurable manifestations of agency such as negotiation); and achievements (including well-being outcomes) [50].

Women’s empowerment is usually operationalized using measures such as women’s participation in household decision-making; access to, or control over household resources (e.g., income); perceptions of gender norms regarding the relationship between couples and gender-based violence; perceived equity in a couple’s power and resources, etc. [54–56, 60]. We do not intend to go into issues of measurement in this paper, as they have been well described elsewhere [51, 55, 60]. However, we describe the role of empowerment using the following broad dimensions: economic, sociocultural, psychological, and cognitive empowerment, which have been used by various authors [51–53, 61, 62].

Women’s economic empowerment is defined as having access to and control over the means to make a living on a sustainable and long-term basis and receiving the material benefits of this access [61]. This includes a woman’s control over income, relative contribution to family support, access to and control of family resources, access to employment, ownership of assets and land, and access to credit, among others [51]. Access to work and income increases economic independence and therefore independence overall [52]. The sociocultural dimension captures gender norms, including norms against gender-based violence, marriage systems, norms regarding women’s physical mobility, discrimination against daughters and commitment to educating girls, women’s visibility in and access to social spaces, and access to modern transportation [51].

Psychological empowerment refers to women believing that they can act at personal and social levels to improve their condition. This is said to involve an escape from “learned helplessness” and the development of self-esteem and confidence [52]. Psychological empowerment captures self-esteem, self-efficacy, and psychological well-being [51]. Stromquist (1995) describes cognitive empowerment as women’s understanding of the causes of their subordination, involving “understanding the self and the need to make choices that may go against cultural or social expectations” [52]. It also includes knowledge about legal rights and sexuality, including family planning [52, 53]. Psychological and cognitive empowerment are closely related, and while they are considered separate dimensions by some classifications [52], only the psychological empowerment is referred to in other classifications [51]. Thus, we combine these two dimensions in this paper.

These dimensions are useful in providing classifications for the numerous ways in which empowerment has been measured in the literature [51]. They also capture resources, agency, and achievements as described by Kabeer [50]. The economic and sociocultural dimensions fall within resources, which Kabeer describes as preconditions to becoming empowered, while psychological and cognitive empowerment tend to fall under agency, which includes processes of decision-making. The outcomes of well-being from any of these dimensions will fall under achievements [50]. In addition, all the dimensions can be operationalized at the individual/household and community levels [51], although some dimensions are measured better on certain levels. For example, the psychological and cognitive dimensions are best measured at the individual level. Economic empowerment could also be measured at the individual or household level. Sociocultural empowerment, on the other hand, may be best measured at the community level. In individual-level analysis of factors associated with prematurity, many of the measures of empowerment will be at the individual or household level. The community-level factors are, however, very important for analysis at any level, and we capture them under contextual factors in our framework.

## Results

### Women’s empowerment and prematurity: direct links

There is very limited evidence supporting a direct link between empowerment and prematurity in developing settings. Our first search in PubMed using all our key terms yielded 2169 articles. Many of these articles were on clinical decision-making, health status, and socioeconomic status in developed settings. We removed status and decision-making from subsequent searches to draw articles that mention empowerment or autonomy. Screening of the titles, abstracts, and full texts yielded 18 articles discussing both empowerment and prematurity [17, 63–79]. These articles were all in developed settings except for one from Iran [75] and a paper from the “Born Too Soon” report on care during pregnancy and childbirth to reduce preterm deliveries and improve health outcomes of the preterm baby, which is a global review [17]. The “Born Too Soon” report references a recent systematic review and meta-analysis which showed that women’s groups practicing participatory learning and action are a cost-effective approach for improving maternal and neonatal health outcomes [80]. However, there are no specific estimates for prematurity in this review. The authors therefore call for more research on how women’s empowerment approaches can translate into reduced preterm birth rates [17].

Thirteen of the articles were on interventions to “empower” parents of premature babies, nine of which were on one program: the Creating Opportunities for Parent Empowerment (COPE) program. These sets of papers showed that the COPE program reduced premature infants’ length of stay in the Neonatal Intensive Care Unit (NICU) and improved parents’ mental health outcomes [63–69, 74, 75]. Our search terms also yielded four articles on empowerment during pregnancy. One of these papers described the use of telephonic nursing, which included case management to support self-care and decision-making, to empower patients at risk for preterm birth [73]. Another was a qualitative study among women hospitalized for preterm labor describing their sense of loss of control and powerlessness and their desire for tools, including information, which would give them a source of empowerment [76]. These studies, however, do not provide evidence that women’s empowerment prevents prematurity or improves outcomes for premature babies. The other studies were on group ANC models, with one describing the comparative effects of group ANC compared to individual care on psychosocial outcomes, and another on the cost-effectiveness of group ANC, with discussions that some of their findings could be attributed to empowerment of women [78, 79]. Other research works in developing settings find that women who receive group ANC are less likely to have a preterm baby compared to women who receive traditional one-on-one care [81–83]. Women’s empowerment is thought to be one of the pathways for some of the observed reduced preterm birth rates in the group ANC models, although this is yet to be empirically evaluated. Centering pregnancy, one of the group ANC models, has been described as a “model of empowerment,” as the model encourages women to take responsibility for themselves, leading to a shift in the client-provider power base [84].

The key dimensions of empowerment captured by these prenatal and postnatal programs are the psychological and cognitive dimensions. While we do not find substantive evidence that women’s empowerment prevents prematurity, these programs do suggest that psychological and cognitive empowerment of women may have a role in improving prematurity outcomes in developed settings [17, 63–79]. Given that women in developing settings tend to be more disadvantaged, “the expansion of their ability to make strategic life choices” [60] may have a large effect on prematurity outcomes. There is a need for studies to provide evidence to support (or refute) this assertion. In lieu of evidence for a direct association between women’s empowerment and prematurity in developing settings, we explored such a link through potential intervening factors—the risk factors for prematurity and the interventions to address it—illustrated in Fig. 1.

### Empowerment and timing of pregnancies: first pregnancy and interpregnancy intervals

There is strong evidence from developing countries pointing to the importance of the sociocultural and economic dimensions of women’s empowerment on delayed childbearing, longer interpregnancy intervals, and lower fertility in general [54]. Sociocultural empowerment is thought to play a role in marriage age, which is associated with fertility and use of modern family planning methods. For example, Abadian (1996) found that women’s age at marriage was inversely associated with total fertility rates [85], while Hogan et al. (1999) found that later age at marriage increased the likelihood of spousal communication regarding family size [86]. While global measures of women’s empowerment have been found to be positively associated with family planning, one study assessing contraceptive use across Africa found that economic decision-making (part of the economic empowerment domain), negotiation of sexual activity, and perceived agreement on fertility preferences (used as measures of familial and interpersonal empowerment domains in that study, but also reflecting psychological and cognitive empowerment as used in this paper) were associated with contraceptive use [87].

There is also a clear link between measures of women’s empowerment and contraceptive use [86, 88–91]. The measure of empowerment used in most of these studies is reproductive autonomy, defined as women’s ability to control and make decisions about contraceptive use and childbearing [54]. This includes freedom from coercion, communication with partners, and decision-making regarding contraceptive use and method choice [91]. Furthermore, a recent systematic review characterizing the domains of empowerment used family planning decision-making and discussions of family planning as measures of women’s empowerment [54]. These measures could be classified more broadly under cognitive and psychological empowerment, suggesting a strong link between cognitive and psychological empowerment and use of contraception. Increased reproductive autonomy suggests that a woman can control when she will use contraception, what method to use, and the decisions regarding her pregnancy, including whether to continue with a pregnancy [92]. Reproductive autonomy may be influenced by a number of factors including individual women’s status, spousal communication and relationship with husbands, and community-level support in contraceptive decision-making [91], thus also reflecting sociocultural empowerment.

In addition to family planning and age at marriage, other studies on women’s empowerment have found that greater household decision-making power is associated with longer interpregnancy intervals [93, 94]. Few studies have, however, assessed specific domains of empowerment and birth intervals. One study in India assessed autonomy using three dimensions: (1) mobility autonomy, (2) household decision-making autonomy, and (3) financial autonomy, which reflect sociocultural, psychological, and economic empowerment, respectively. The study found that women with higher levels of autonomy had higher birth-to-conception intervals compared to women with lower levels of autonomy [95]. Since several dimensions of empowerment are associated with delayed first birth, longer interpregnancy intervals, and lower parity, and these factors decrease the risk of prematurity, it is plausible that empowerment will decrease the risk of prematurity.

### Empowerment and nutritional status

Measures of women’s empowerment are also strongly related to women’s nutritional status as well as that of their children [96–98]. Studies in developing settings have shown empowerment measures are positively associated with calorie availability and dietary diversity at the household level as well as better maternal nutrition [97–99]. Studies examining empowerment and nutritional status use various measures of empowerment, one example being the Women’s Empowerment in Agriculture Index, which is an aggregate index including five domains of empowerment (agricultural production decisions, access to and decision-making power over productive resources, control over use of income, leadership roles within the community, and time allocation) [100]. These domains reflect empowerment across the dimensions we use in this paper. While some studies have found overall empowerment is positively associated with better maternal nutrition [97], others find significant effects for only the domains related to economic empowerment [98].

Economic empowerment creates the resources for acquiring healthy foods in good quantities, quality, and diversity [98, 101, 102]. Cognitive empowerment, on the other hand, may equip women with the knowledge to select healthy foods for themselves and their households. Evidence from SSA suggests that women’s control of resources is linked to larger allocations of resources to food [103, 104]. Women’s empowerment has also been found to mitigate the negative effect of low agricultural production diversity on maternal and child dietary quality; i.e., even when agricultural households produce only a few food groups, the quality of the diet of women and their children, as measured by the diversity of the foods consumed, is improved if women score higher on measures of empowerment [97]. This evidence implies that women’s empowerment will increase the chances that women enter pregnancy in good nutritional status and maintain good nutritional status during pregnancy, decreasing their risk for preterm birth.

### Empowerment and psychological health

**Fig. 2:**
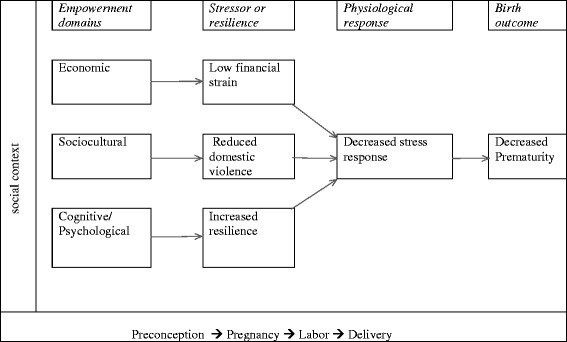
Empowerment, psychological health, and prematurity pathways

While there is little evidence linking women’s empowerment and stress in developing settings, the relationship between stress (and maternal psychological health in general) and prematurity is very strong [1, 28, 32–34], and the relationship between women’s empowerment and stress is plausible for several reasons. We therefore highlight potential relationships between various dimensions of women’s empowerment and psychological health (see Fig. 2), emphasizing the known links between stress and prematurity, and proposing other links that can be empirically tested. The model shows that economic empowerment will decrease prematurity by lowering financial strain as a source of stress. On the other hand, a decrease in domestic violence, which may result from increased sociocultural empowerment, would decrease prematurity. We also posit that cognitive/psychological empowerment affects the stress response by building women’s resilience to cope with stress. These relationships are plausible for the following reasons.

First, acute, episodic, and chronic stress are all hypothesized to emanate from inequities in determinants of health such as education, income, employment, nutrition, social support, and access to health care [105, 106]. Disempowerment increases inequities in these health determinants. Women’s empowerment, conversely, may help mitigate the effects of these factors on the stress experienced by women. In particular, economic empowerment may reduce or eliminate financial strain as a stressor [39, 107]. Psychological empowerment, on the other hand, may contribute to resilience through ego-related resources such as mastery or self-efficacy, perceived control, and self-esteem, which can decrease the negative effects of stress on birth outcomes [35, 108]. A woman’s confidence in her ability to act at personal and social levels to improve her condition may also enable her to confront authorities, when necessary, to defend her rights and obtain necessary auxiliary medical and social services, including psychological services, to help her cope with stress—potentially improving her birth outcomes [52].

Second, women who experience any form of domestic violence are twice as likely to give birth to a premature baby, and one of the mechanisms for this association is thought to be through increased maternal stress [38]. Almost half of the women reporting serious domestic violence also meet the criteria for major depression; 24% of these women suffer from posttraumatic stress disorder, and 31% from anxiety [109, 110]. However, the relationship between stress and domestic violence can be bidirectional, with stress leading to domestic violence and, conversely, domestic violence creating stress. Stress has been identified as a cause of domestic violence, especially economic stress and strain [111], and violent behavior has been proposed as a likely behavior among people with particular methods of evaluating and coping with stress [112]. Equally, domestic violence is a cause of stress, as women who suffer domestic violence experience overwhelming mental and emotional distress [109].

There is also indirect evidence that women’s empowerment, especially economic empowerment, decreases both domestic violence and stress [113, 114]. The proposed mechanism is that women’s economic, sociocultural, and psychological empowerment will nurture their inner strength, creativity, and self-esteem and build resilience, all leading to stress reduction [115]. Equipping girls and women with access to economic resources and the power to make decisions for themselves and their families has been shown to indirectly reduce violence against them [116, 117]. The evidence for the role of economic empowerment in reducing domestic violence from developing settings is, however, still limited, and results are mixed [118, 119]. These mixed results may be due to the important role of context and social norms [115, 119]. Sociocultural empowerment, particularly related to gender norms about intimate partner violence, therefore has strong potential for reducing domestic violence and the associated stress [120].

That measures of women’s empowerment are associated with domestic violence, which is associated with stress, therefore increases the plausibility of a relationship between empowerment and prematurity. Furthermore, stress is only one of the pathways by which domestic violence may affect prematurity, as much is yet to be learned about the factors underlying the effect of violence on prematurity. Other studies show women who experience domestic violence are less likely to use health services during pregnancy [121]. This may be a confounding effect of the underlying factors like disempowerment across other domains, but it may also suggest an effect of the violence/stress pathways on health care-seeking behaviors.

### Empowerment and access to good quality maternal health care

As discussed above, pregnancy and childbirth are critical windows of opportunity to provide effective interventions to prevent prematurity and improve survival and development of premature babies. Yet, significant gaps in coverage, equity, and quality of prenatal and delivery care remain between and within countries [1, 17]. Measures of women’s empowerment have been found to be important predictors of the use of maternal health services in developing settings.

The barriers to the use of maternal health services in developing settings include lack of control over the material resources needed to pay for expenses, poor physical accessibility, poor perceptions of care in health facilities, and sociocultural factors including cultural norms regarding women’s mobility and beliefs about the causes of difficult labor that influence perceived need for services [122–124]. Women’s lack of control over the decision to seek care is also a barrier to use of services, especially in communities where women cannot go to the hospital without the permission of their husbands or other family elders—even when there is an obvious need for hospital care [125–127]. Women’s empowerment across various dimensions helps to mitigate the effects of some of these barriers [122, 123, 128–130].

Most studies suggest women’s empowerment increases the use of ANC and delivery in health facilities (or with skilled birth attendants) [56, 122, 123, 131–134]. These studies use empowerment measures that capture the economic, sociocultural, psychological, and cognitive dimensions of empowerment, and most studies find significant associations for at least some dimensions, although the important dimensions vary from study to study [123, 129, 130, 134–139]. Economic empowerment enables women to overcome financial barriers to accessing services. Economic empowerment may also help to mitigate the effects of physical access, as women with the means are able to pay for the costs of travel to reach health facilities. Education and wealth are the most often used measures for economic empowerment, and these are consistently associated with higher utilization of maternal health services in many settings. In addition, some studies find positive associations between use of health services and measures of financial independence and access to, or control over, household resources [56, 123, 132, 134].

The effect of sociocultural empowerment is reflected in the associations between use of health services and measures of women’s freedom of movement and perceptions of gender norms [122, 123, 128]. Not many studies examine measures related to psychological and cognitive empowerment and use of maternal health services in developing settings. However, a few studies have found that women with more knowledge of pregnancy complications, which could be considered a measure of cognitive empowerment, are more likely to use services [140–143]. Participation in household decision-making, a common measure used in studies of use of maternal health services, could also be considered a measure of psychological empowerment, although it might also reflect economic and socio-cultural empowerment. Several studies, including a meta-analysis on the use of skilled birth attendants in 31 countries, found that participation in household decision-making was positively associated with the use of maternal health services [128, 130, 136, 137]. The effect, however, may be contextual, as other studies found no significant associations between participation in household decision-making and the use of maternal health services in some settings [135, 138, 144].

There is less empirical evidence on women’s empowerment and quality of care, as the traditional measures of empowerment have not been used to predict quality of care. However, the role of empowerment could be implied from the studies that examine education and wealth as predictors of quality. These studies show that women of higher economic status receive better quality of care—as measured by services received (technical dimensions of quality) as well as by reports of women’s experiences of how they are treated in health facilities (interpersonal dimensions of quality)—from preconception, including family planning, to care during pregnancy, delivery, and after delivery, as well as care of the newborn [145–155]. Other studies suggest poorer women and those with low education are more likely to be disrespected and treated poorly in health facilities [147, 149–151]. More empowered women may be more likely to receive better quality care because they have the economic resources to afford this level of care and have the skills to interact with providers and navigate the health care setting to demand better quality of care [145].

## Discussion

This paper highlights four potential pathways by which women’s empowerment could potentially reduce the burden of prematurity, through factors that are already known to affect prematurity. The conceptual model (Fig. 1) shows that women’s empowerment may reduce prematurity by (1) preventing early marriage and promoting family planning, which will delay first pregnancy and increase interpregnancy intervals; (2) improving women’s nutritional status; (3) reducing domestic violence and other stressors and increasing resilience; and (4) promoting receipt of good quality care during pregnancy and delivery. Women’s empowerment is thus a distal factor that affects prematurity through a number of intermediate factors. These hypothesized relationships are the relationships for which we found some evidence—both direct and indirect—in the review of the literature. They are intended to be an illustrative set of relationships, rather than an exhaustive set.

Although we discussed in our review of the literature how the different dimensions of empowerment might affect the intervening factors we propose, for parsimony we do not specify these dimensions in the primary conceptual framework. This is, however, illustrated with the stress pathway in Fig. 2. We also do not show the relationships between the intervening factors, some of which we have discussed above, to avoid having too many arrows in the diagram. In addition, the pathway by which each dimension affects the intervening factors in our models may differ for different dimensions of empowerment. For example, for the relationship between empowerment and use of maternal health services, economic empowerment likely improves use by increasing physical and financial access to services, while psychological and cognitive empowerment increases perceptions of need for care. However, sociocultural empowerment may be more important in settings where the main barriers to use of services are from cultural factors that prevent women from using the services [123, 124, 127]. These nuances, though important, are too many to highlight in the diagram but need to be considered in its application.

We show in the conceptual model that the relationship between empowerment and prematurity depends on the context and spans the life course, starting before pregnancy and lasting until after delivery. The contribution of the different dimensions of empowerment may, however, be different at different times in the life course. For instance, sociocultural empowerment may be especially important for preventing adolescent pregnancies due to early marriage, as this is influenced by norms around early marriage and also gender norms around female education. For older women, cognitive and economic empowerment may be more important for increasing interpregnancy interval through the decision to use contraception as well as getting access to the service. The effect of women’s empowerment could also be across generations. The empowerment of women across different dimensions will increase the chances that their female children stay in school and do not marry early, thus reducing the chances of their children having premature babies. Women’s empowerment could also decrease adverse childhood experience among their children (as domestic violence against women is also traumatizing for their children), decreasing their future risk for stress-related prematurity [37].

While there is evidence to support most of the pathways we posit, there are several gaps in the literature.

The first gap in the literature is the almost absent evidence on a direct association between women’s empowerment and prematurity. Given the increasing interest in women’s empowerment on one hand and prematurity on the other, there is a need for research linking the two to help push the women’s empowerment agenda among unlikely allies. Future research should focus on empirically demonstrating the links between women’s empowerment and prematurity. In addition, even though there is good evidence linking early childbearing and interpregnancy intervals, women’s nutritional status, and stress to prematurity, more studies are needed in developing settings to strengthen the recommendations on use of services, as this recommendation is based mostly on evidence in developed settings and on assumptions on quality of care, which are often not met.

Among the intervening pathways we propose, there is substantial evidence on the association between women’s empowerment and three of the intervening factors: early childbearing and interpregnancy intervals, women’s nutritional status, and use of services. Nonetheless, more research is needed on the “how” of these associations for the different dimensions of empowerment. Even though the literature on empowerment and family planning, nutrition, and use of maternal health services is robust, few studies have empirically examined the intervening pathways by which different dimensions of empowerment affect these intervening factors. There is also limited evidence on if and how women’s empowerment affects the quality of care women receive. Examining the factors that mediate the association between different dimensions of empowerment and the intervening factors is, however, important for interventions, as the mediating factors may vary by contexts. Potential limitations in the literature include a lack of consensus on how to measure women’s empowerment, different domains used across studies, and lack of robust data to explore potential pathways. Future research on the individual intervening factors should therefore examine in more detail the pathways by which different dimensions of empowerment influence each of the intervening factors to affect prematurity.

Among the relationships proposed, the pathway with the least evidence is the empowerment stress pathway. Very few studies have examined the relationship between empowerment and stress in developing settings, and even fewer studies have examined how empowerment might affect women’s stress response in developing settings. This is likely due to a separation in the fields that have an interest in empowerment and those with an interest in stress or psychological health in general. However, more studies are needed in this area, especially in developing regions, given the significant evidence between stress and prematurity, and the disempowerment of women in these settings. To stimulate the discussion on empowerment and stress, we have highlighted the empowerment stress pathway in Fig. 2. The diagram is intended to be a skeletal framework with pathways that can be tested through empirical analysis. We do not attempt to expand on the physiological pathways between stress and prematurity; this has been examined in other work [28, 32]. We are also likely missing other important stressors, which we hope will be identified from more research on the topic. Our goal is to stimulate a discussion across disciplines. Research on empowerment and stress is one of the areas that will benefit greatly from transdisciplinary collaborations.

In addition, we focus on empowerment as the distal factor in this framework. However, empowerment is also a potential moderator; for example, empowerment may moderate the relationship between stress and prematurity. Disentangling these complex relationships will help inform future interventions to reduce prematurity. Finally, empowerment is not a static construct, but rather can change over time. Across the life course, women may be more or less empowered depending on a number of factors, e.g., their current life situation and life events, their family and household dynamics, and their communities and institutions in which they are embedded. A better understanding of how empowerment may change across the life course and the influence on different health outcomes, including prematurity, will be important for interventions targeting women and families.

This manuscript raises several unanswered questions needing empirical answers, which include: Does empowerment have a direct effect on prematurity net of the known risk factors? Does women’s empowerment moderate the effects of various risk factors? Which dimensions of empowerment are important for prematurity? How does empowerment affect prematurity in different settings? And will interventions targeting women’s empowerment help reduce prematurity and improve survival of premature babies? We hope suggesting some of the potential links will stimulate more research on empowerment and prematurity. Our framework also has implications for causal inference. For example, what kinds of biases will be inherent in estimating the effect of empowerment on prematurity via some intervening factors, if other factors are not considered? Researchers need to carefully think through these issues in the application of this framework—as in all research. Like all conceptual frameworks, our framework is a tool to help organize our ideas around empowerment and prematurity, and the relationships we posit may have variations in different contexts. More research is needed to flesh out each of the pathways and potential connections between the intervening factors in different contexts. While this work is focused on prematurity, the framework could also apply to other birth outcomes, including low birth weight and stillbirths, as these tend to have overlapping determinants [156–159].

A limitation of this paper is that some of the pathways in our conceptual model are based on plausible links without substantive empirical analysis to support them. However, our intention is that this paper will stimulate research to substantiate the pathways or determine alternative pathways that can be acted upon to both empower women and reduce the burden of prematurity. As with any review of the literature, studies are prone to publication bias, suggesting that these findings may represent overestimation of effects and associations between empowerment and potential pathways of prematurity. However, we attempt to look across contexts and studies to identify and summarize relevant conclusions. We also focus on developing countries, because the predominant factors affecting prematurity are different between developed and developing countries. Future studies could focus on developed countries, including examining issues of race, intersectionality, and other relevant issues in the developed context. Country-level analysis of trends between measures of gender equality and preterm birth over time might also extend the evidence for the empowerment prematurity relationship.

## Conclusions

The determinants of prematurity are varied and complex. We posit that women’s empowerment is a distal factor that may affect many known risk factors of prematurity, as well as the recommended mechanisms to address prematurity. This paper proposes a conceptual model that illustrates pathways by which women’s empowerment may decrease prematurity, focused on four intervening factors. Conceptual models are useful in guiding research and programs, because they provide a visual representation of theoretical and important domains of interest and provide a common basis for researchers and policy-makers to discuss and understand different mechanisms and pathways involved in any relationship of interest. Given advancements and interest in women’s empowerment on one hand and prematurity on the other, this conceptual model provides a roadmap for research linking women’s empowerment and prematurity, and it lays the foundation for researchers to be able to test different mechanisms and the ways in which constructs may mediate, moderate, or affect one another in the empowerment prematurity pathway.

## Open peer review

Peer review reports for this article are available in Additional file 1.

## Additional file

Additional file 1: Open peer review. (PDF 223 kb)

## Abbreviations

ANC: Antenatal care
COPE: Creating Opportunities for Parent Empowerment
NICU: Neonatal Intensive Care Unit
PPROM: Prelabor premature rupture of membranes
SSA: Sub-Saharan Africa
