# Supplementary material for: Conceptualizing pathways linking women’s empowerment and prematurity in developing countries
Source: BMC Pregnancy Childbirth. 2017 Nov 8;17(Suppl 2):338. doi: 10.1186/s12884-017-1502-6 (PMC5688445; doi:10.1186/s12884-017-1502-6)
Supplement: Additional file 1: — Open peer review. (PDF 223 kb) [file 12884_2017_1502_MOESM1_ESM.pdf]

Reviewer reports

Title: Conceptualizing pathways linking women's empowerment and prematurity in developing countries

Reviewer 1: Corrina Moucheraud

Reviewer's report

I feel that this is an interesting and important topic, and I think this manuscript is thoughtful and well-written. That said, I feel the manuscript needs some improvements before it can be accepted for publication. My main concerns about this paper are the selection of the 4 "intervening factors" and the hypothesized relationships between the variables, as described below.

- Major Compulsory Revisions

1. The authors must provide a justification for selecting these 4 "intervening factors." Without such a rationale, it is impossible for the reader (or reviewer) to assess whether all, or even most, important variables are included. As a theory-building, hypothesis-generating paper, I feel it is essential to justify choices such as this one.

2. I understand that this was not a systematic review – which likely would be necessary for developing a "definitive" conceptual framework – but nonetheless I feel the authors should provide more information about their literature review. What databases were searched – only peer-reviewed literature, or the grey literature too? And in what disciplines? Were there any date, language, or regional restrictions? Were there other exclusion criteria? How many articles were reviewed? Again, given that the objective of this paper is to inform/guide future empirical analyses, it's important for the reader to know this so they can develop an informed opinion about whether they trust & want to use this framework.

3. The authors should provide more information about how they developed the arrows (hypothesized relationships) between constructs in Figure 1. For example, I would argue that there is likely a relationship between domestic violence and accessing quality maternal health care, but there is no such arrow in Figure 1.
Is this intended to be an exhaustive set of relationships, or is it simply illustrative? If the former, the authors need to demonstrate the methods used to establish these. If the latter, they need to more clearly document their research methods so a reader can draw their own conclusions about possible omissions etc.

4. It also seems that many other possible relationships are not represented in Figure 1, including some mentioned in the manuscript itself – including the possibility of a direct link between empowerment and prematurity, and "empowerment may moderate the relationship

between stress and prematurity" (both mentioned in the Discussion section, 5th and 6th paragraphs).

5. Lastly, I encourage the authors to think about how their proposed framework (Figure 1) might affect empirical work, particularly from a causal inference perspective. For instance, the hypothesized link between "reduce adolescent pregnancies & increase IP intervals" and "improved nutritional status" means that any attempt to estimate the effect of empowerment and prematurity, via nutritional resources, would be inherently biased if family planning was not included (i.e., it would introduce omitted variable bias).

- Minor Essential Revisions
1. I suggest that, in Intro para 1, the authors note whether the change from 7.5% to 8.6% was a statistically significant increase.

2. I think that it's important for the authors to clarify why they focused on preterm birth – rather than, for example, small for gestational age/low birthweight which, one could argue, is more inclusive & circumvents some of the methodological challenges with calculating preterm births, and is likely relevant to the empowerment variables discussed here.

3. In the paragraph beginning "In the preconception period family planning is a highly cost-effective intervention" – note that family planning is, in itself, not an intervention. I would suggest rephrasing this.

4. I feel that the first paragraph within "Is there a role of women's empowerment" (beginning "Little is known about how women's empowerment is associated with prematurity") is a result of this analysis, not background information – and should be placed in that section accordingly.

5. The first paragraph within Results says that "The only relevant articles from a search on empowerment and prematurity concern research on interventions to 'empower' parents of premature babies in developed settings" – but the second example given (beginning "Recent work in developing settings on group antenatal care (ANC)…") sounds highly relevant so I suggest a rephrase, or else a clearer description of why this is not a valuable example.

- Discretionary Revisions
1. I feel it would be helpful for the authors to somewhere mention stillbirths, as these are closely linked to preterm.

2. In the "Recommended interventions to address the burden of prematurity" section, I am unclear about why certain phrases are italicized. If this is meant to refer to steps along the continuum of care, I would suggest doing this consistently (i.e., what's the relationship between "preconception period," "antenatal care," and "skilled attendance"?).

Level of interest: An article whose findings are important to those with closely related research interests

Quality of written English: Acceptable

Declaration of competing interests: I declare that I have no competing interests

Reviewer 2: Kirsten Stoebenau

Reviewer's Report

Overall, I found the paper "Conceptualizing pathways linking women's empowerment and prematurity in developing countries" an interesting and thought-provoking contribution. The authors clearly state the overall aim of the paper, grapple with the dimensions of women's empowerment, and provide important understanding of the known causes and recommended interventions for preterm birth. The review of the relationship between the different dimensions of empowerment and preterm birth and related outcomes could be presented with greater clarity. I provide a number of recommendations toward strengthening the clarity of the presentation and its arguments below toward further strengthening this contribution to the field of maternal and child health.

Major Compulsory Revisions

1. Organization of the paper – I found the arguments at times very hard to follow; and felt that explanation for approaches the authors chose to make came too late, and left too much room for me to question their methods, approaches or decisions. The paper could be significantly strengthened by re-organizing the presentation of some of the sections and where and how the authors introduce different points. There are multiple examples of this. Most importantly:
    a. The presentation of the methods section should precede the discussion on empowerment. We need to understand how you came to learn about empowerment and how it relates to preterm birth. The reader will trust you more if you have already taken us through the steps of your review before you present your conceptualization of empowerment.
    b. I would recommend an introduction which includes a review of definition and epidemiology of preterm birth, quickly describes how this differs in high income versus low income settings and then clearly focuses the reader to the low income setting; follow this with methods and then lay out the conceptualization of empowerment for the purposes of understanding preterm birth.
    c. Presentation of the intervening factors/outcomes that you link to empowerment. Perhaps spend more time on introducing the reader specifically to first pregnancy, spacing, nutrition and stress up front, before you then conceptualize how these link to empowerment. This way the reader will already be convinced of their role in prematurity/preterm birth and then can focus on how empowering women across different dimensions can then improve these outcomes and in turn impact preterm birth. It would also help to include in the methods section how you came to focus on these topics and how you searched for articles on these topics, specifically.
    d. Presentation of the figures. It would strengthen the paper if the figures were presented ahead of their being described. Figure 1 could be introduced ahead of your presenting the results for each of these outcomes. This would orient the

reader. Through each section you can flesh out how different dimensions and sub-domains of empowerment lead to improved intervening outcomes and then on to impact preterm birth.

e. The presentation of Figure 2. Presenting Figure 1 upfront would then allow you to present Figure 2 when you actually describe the hypothesized linkages between different dimensions of empowerment and stress. For this- State up front that while there is little literature connecting different indicators or measures of empowerment to stress, that findings connecting stress to preterm birth are very strong, and there are plausible links between different dimensions of empowerment and stress, therefore, you will emphasize these links and propose a set of linkages that can then be empirically tested. Make sure this is clear up front – don't want for the discussion section to tell us why you focused on it. This will keep the reader with you.

2. Conceptualizing Empowerment.
   a. The use of Kabeer is great; it's well-used and well-respected. One place where I see some of your application of Kabeer's work shift from how I have interpreted it, at least, is that you seem to consider empowerment as a *factor*. Kabeer describes it as a *process* that can be conceptualized across several domains each containing sub-domains. There are factors that facilitate empowerment processes. My biggest concern along these lines makes up my next set of comments:
   b. Socio-cultural Empowerment – I simply disagree with how you have framed socio-cultural empowerment. (first paragraph, page 8) For me, many of the aspects that you have captured under socio-cultural empowerment are enablers or facilitators of empowerment, but not dimensions of empowerment themselves. Specifically, gender norms, marriage systems – these are hugely important contextual determinants that when changed can transform a context into one where women can become empowered (or not). Perhaps indicating that these sub-domains fall within 'resources,' or as Kabeer describes resources, the "pre-conditions" to becoming empowered, would help with this. I also think, though, that because the outcomes you are addressing in this paper are very much at an individual level, then there needs to be some recognition of distinctions between community-level indicators versus individual ones. This becomes an issue in your figures where the distinctions between more macro and micro-level processes are not very clear. I just find this 'dimension' would serve better conceptually as a set of contextual factors (as you have indicated in your diagram – what does 'social context' constitute if not these very issues that you are defining as 'socio-cultural empowerment?')
   c. It would also help if you could provide a rationale for the specific dimensions of empowerment you choose to focus on (economic, sociocultural, psychological,

and cognitive). Why these, specifically? Have they been explored by others and in this way? (I know the answer for most of these is emphatically yes, but this just needs to be explained, that said, I am not familiar with 'cognitive empowerment,' especially not as it might differ significantly from 'psychological' empowerment.)

   d. Perhaps if you draw on Kabeer more closely you could describe socio-cultural empowerment and cognitive empowerment as 'resources' at the community and individual levels, respectively? Somehow the more collective versus individual level dimensions need to be differentiated in the text and ideally in the figures, if possible.
   e. Distinguishing Autonomy from Empowerment - Autonomy is often used as an indicator for empowerment, especially in the South Asian context where it's understood that it is often denied to women, but be careful not to equate autonomy with empowerment, or use them interchangeably. It might help to define it as well, which I don't think you do.

3. Methods - I find the current description of the methods section inadequate. What databases did you search? How many articles did you find, approximately, that fit the topic? What key words did you use to identify articles on preterm birth? How did you select these? How did you organize the literature that you found? How did you settle on the intervening factors you describe at the top of page 12 in the results section, as those that would then be the focus for this paper?
   a. Methods in abstract – the language you have in the abstract for methods is not 'methods' to me. It's more a description of your findings. Once you expand the 'how' in the body of the text please use that to guide what you include for the Methods section in the abstract.

4. Findings – Throughout this section I would encourage you to include more specific language linking specific pathways.
   a. Stress – as one example, for stress, is there evidence for any of the proposed linkages you cite between different domains of women's empowerment and stress that you can lay out more specifically? For example – economic empowerment: how does employment, or land ownership or escaping poverty, relate to stress? Are their studies that look at different measures of self-efficacy, confidence, decision-making and stress?
   b. Services: In this section, I became concerned with what seemed like an assumption that an empowered woman would choose to use health services (last lines of first paragraph). I believe Kabeer cautions about this– I think you have to be a little careful in assuming that an "empowered woman" is going to make the choices that you as healthcare researcher/advocate/provider would like her to make. An empowered mother may choose to marry her daughter before the age of 18; an empowered woman may autonomously choose to

deliver her baby at home; exercising agency/self-efficacy. This might help explain how some studies don't find expected linkages between decision-making power, or other forms of autonomy and health service use (as you state on page17, second paragraph).

c. On specific language and cognitive empowerment – While knowledge is power, it would strengthen your argument when you describe cognitive empowerment if you were to delineate how the different sets of knowledge you code as cognitive empowerment will then improve a woman's ability to stay healthy and have a healthy baby. In other words- how would these specific bodies of knowledge get transformed into action (which would be more indicative of being empowering)?
d. Quality of Services- I am not sure this section should be included. There is such a large body of evidence around the relationship between wealth / education and health. We know that wealthier people are healthier and can afford better health services. I think this could be a sentence or two rather than an entire section as the pathway is well worn. Perhaps leave this to the discussion section?

Minor Essential Revisions

1. Throughout, make sure you cite your sources. You provide examples of psychological empowerment right out of Malhotra et al, but don't cite it there. You have quotes with no citations following them. Check this throughout.
2. The opening paragraph of the introduction (page 3) is a bit confusing as it is currently laid out for me, and this may be because I have spent time considering the issue of fertility decline, specifically. I understand that the intention is to demonstrate that preterm birth is a problem and it's becoming an increasingly serious problem. However, the authors suggest first that preterm birth is more problematic in LMICS than HICs, and suggest that this is probably due to high fertility. Then, the authors suggest that trend data suggest that preterm birth is rising, and cite Latin America as an example- but Latin America has experienced massive, rapid fertility decline, which would suggest that if fertility levels explain higher preterm birth then one would expect the rate of preterm births to be falling, not rising. I think this opening paragraph needs to be stronger, and perhaps should not begin with efforts to explain causality, but simply state what we know about preterm birth rates/trends.
3. Fix the use of the word "dimensions" with respect to empowerment on line 129 (third paragraph, page 7) – as you describe other sets of dimensions again on lines 13-138 (first paragraph, page 8). Maybe "components" for line 129?
4. Emphasis on Life Course – I would just move this to the Discussion and not suggest up front in the introduction that you will use lifecourse as a specific lens through which you review this literature. It's mentioned far too infrequently for that. The lines between 431 and 438 (second paragraph page 19) seem to come out of nowhere.

5. Use of the word 'proxy'. I am a bit struck by your use of the word proxy for different measures of empowerment. As a 'proxy' is a 'in lieu of what we'd like to use, we use this' notion, it suggests that we are not able to measure empowerment. As you indicate, there have been MANY efforts to build indices and scales and novel methodologies to try to capture empowerment. I think in many cases you could just describe different 'measures' that capture different dimensions of empowerment, rather than calling them all 'proxies'. In my mind, the more direct measures get at women's control and decision-making, negotiation and address whether and how these have changed over time. The less direct, more appropriately described as 'proxy' measures, are those that measure 'acheivements' alone without an understanding of a woman's role in getting to those achievements; or a sense of how/whether they will be able to benefit her life and ability to make choices to improve it. So, education, income, labor force participation; these to me are more 'proxy' measures than say decision-making, control over resources etc. Mostly, though, I find the repeated use of the word proxy suggests that we have no way of actually capturing empowerment.
    a. Lines 132-135 (1st paragraph, page 8): some of the measures you cite as proxy measures here are, in my mind, some of the more direct measures we have (e.g. negotiation/decision-making [if these were change measures])
    b. Lines 354-355 (3rd paragraph, page 16) – maybe just "measures"
    c. Line 360 (3rd paragraph, page 16) "education and wealth" are not the BEST proxies, they are among the most often used, because they are among the most often measured.
6. Use of term "empowerment" throughout the paper:- You have done a nice job of showing the reader how complicated and multifaceted 'empowerment' is in the lead up to the presentation of the results; but then don't always maintain this complexity in the presentation of the findings. Please just continue to qualify "empowerment" with phrases like "dimensions of empowerment" or "different measures of empowerment" to maintain this important nuance/complexity.
    a. For example, line 216 – "women who are empowered" … You go on to describe the different dimensions of empowerment, specifically, but it might be stronger if you started with that level of specificity. (e.g. - There is evidence pointing to the importance of the socio-cultural and economic dimensions of women's empowerment in determining outcomes for timing of first birth and interbirth intervals).
    b. Another example – line 254 "since empowerment is associated with… " … "it is plausible that empowerment will decrease…" Again, it would be more powerful if you were more specific about which dimensions you had reviewed, or indicated.
7. Findings - Connecting sentences from one section to the next: (e.g. line 258-259) I don't think you need to bring the reader to the next heading like this, I would just finish the

topic and then use a new heading to introduce the next. You have already highlighted above that there will be four sets of findings.

8. I admittedly know very little about the topic of preterm birth, but the role of technology and access to advanced medical equipment that comes with wealthy country contexts seems incredibly important with respect to the differences in mortality levels from preterm births in LMICs as compared to HICs. If this is the case, I think it should be mentioned. This seemed to be a particularly obvious omission on page 4 paragraph 2 where the focus is on the importance of at least a TBA in reducing morbidity/mortality risk associated with prematurity. Perhaps I am too focused on the extreme case (births at 25 weeks); but it would help a lay reader if the authors could explain what the majority-case preterm birth looks like and then how TBAs rather than fully equipped NICU's, can change the outcomes of these births.
    a. Related, as you are focused on the developing country context, make sure your examples reflect that reality. I wasn't sure on Table 1 if among the causes it was necessary to highlight "increased rates of twins and higher order pregnancies with assisted reproduction." Keep the reader focused on the context you have in mind.
9. Fix the sentence on lines 38-39 (last sentence paragraph 1, page 4). As stated, it doesn't make sense.

Minor Discretionary Revisions

1. Discussion - Quality of services - that the quality of care necessary to reduce mortality among preterm birth babies is, I suspect (I don't know the literature) lacking in the developing country setting seems like something that should be addressed more thoroughly; and makes me wonder if prevention is therefore even more important if that would help save more lives if and until better quality of care of is available to the majority of the population in many lower income country settings.
2. One thought I had for a future research project on this topic would be a country-level analysis of the trends between measures of gender equality and rates of pre-term birth, over time.

I'd like to thank the authors for their hard work. I really enjoyed reading this paper. It pushed my thinking and taught me a lot. Thanks so much.

Level of interest
- An article whose findings are important to those with closely related research interests

Quality of written English
- Needs some language corrections before being published (Needs quite a few.)

I declare that I have no competing interests.

Response to reviewers

Reviewer Comments	Response
	Thank you for your insightful comments and suggestions, we have edited the manuscript to address them as best as we can and responded to them below. They have greatly improved the paper.
**Reviewer #1**	
I feel that this is an interesting and important topic, and I think this manuscript is thoughtful and well-written. That said, I feel the manuscript needs some improvements before it can be accepted for publication. My main concerns about this paper are the selection of the 4 "intervening factors" and the hypothesized relationships between the variables, as described below.	Thank you. We have tried to address them as best as we can and responded to them below
-Major Compulsory Revisions	
1. The authors must provide a justification for selecting these 4 "intervening factors." Without such a rationale, it is impossible for the reader (or reviewer) to assess whether all, or even most, important variables are included. As a theory-building, hypothesis-generating paper, I feel it is essential to justify choices such as this one.	We have provided justification for the selection of these factors in the section we present them. We have also expanded the discussion on these factors to highlight why we think these are important factors.
2. I understand that this was not a systematic review – which likely would be necessary for developing a "definitive" conceptual framework – but nonetheless I feel the authors should provide more information about their literature review. What databases were searched – only peer-reviewed literature, or the grey literature too? And in what disciplines? Were there any date, language, or regional restrictions? Were there other exclusion criteria? How many articles were reviewed? Again, given that the objective of this paper is to inform/guide future empirical analyses, it's important for the reader to know this so they can develop an informed opinion about whether they trust & want to use this framework.	While this was not meant to be a systematic review, we have provided more details on the literature review in the methods section; and of the articles reviewed in the first part of the results section. Thank you for your guidance on this aspect of the manuscript.
3. The authors should provide more information about how they developed the arrows (hypothesized relationships) between constructs in Figure 1. For example, I would argue that there is likely a relationship between domestic violence and accessing quality maternal health care, but there is no such arrow in Figure 1.<br>Is this intended to be an exhaustive set of relationships, or is it simply illustrative? If the former, the authors need to demonstrate the methods used to establish these. If the latter, they need to more clearly document	We have provided more information on this in the section on the intervening factors, and in the discussion to emphasize that these are plausible linkages we identified based on our prior knowledge and then searched the literature to see if the evidence supported it.<br><br>We note that<br>"These hypothesized relationships are the relationships we found some evidence for in the review of the literature—both direct and indirect. They are intended to

their research methods so a reader can draw their own conclusions about possible omissions etc	be an illustrative set of relationships, rather than an exhaustive set."<br><br>We agree that there is likely a relationship between domestic violence and accessing quality maternal health care and we do mention this. In addition we discussed other possible relationships between the intervening factors in the discussion of the risk factors or where appropriate in the links between empowerment and the intervening factors. However we had chosen not to add arrows for all these potential relationships because of comments on an earlier version that suggested the too many arrows were distracting and so to focus on the key relationships in our pathway and only discuss the other potential pathways. We have removed the arrows between other intervening factors (relationship between adolescent pregnancies and stress and improved nutritional status) to be consistent. We have specified that these arrows are missing from the diagram.<br><br>We have also expanded on the methods to allow readers to make their own conclusions about possible omissions.
4. It also seems that many other possible relationships are not represented in Figure 1, including some mentioned in the manuscript itself – including the possibility of a direct link between empowerment and prematurity, and "empowerment may moderate the relationship between stress and prematurity" (both mentioned in the Discussion section, 5th and 6th paragraphs).	Yes, we acknowledge that there are many other possible relationships not described in this manuscript. As mentioned we were advised in a earlier version to avoid too many arrows in the diagram because they were distracting, and removing some of these arrows did simplify the figure to bring out the essential relationships from empowerment to the intervening factors to prematurity. We have discussed these other potential relationships so they can be considered in future research. We have also added that like all conceptual frameworks, this is only a tool to help organize our ideas around empowerment and prematurity and may have variations in different context.
5. Lastly, I encourage the authors to think about how their proposed framework (Figure 1) might affect empirical work, particularly from a causal inference perspective. For instance, the hypothesized link between "reduce adolescent pregnancies & increase IP intervals" and "improved nutritional status" means that any attempt to estimate the effect of empowerment and prematurity, via nutritional resources, would be inherently biased if family planning was not included (i.e., it would introduce omitted variable bias).	We have added a sentence in the last paragraph of the discussion before the limitations to highlight this. Thank you for your suggestion.
-Minor Essential Revisions	
1. I suggest that, in Intro para 1, the authors note whether the change from 7.5% to 8.6% was a	It is unclear in the report if the change in the rate is significant, but there is an overlap in the confidence

statistically significant increase.	intervals for the absolute numbers of preterm births in the two time periods provided in the paper:<br><br>*"The mean estimated rate in these countries for 1990 was 7·5% (total preterm births in these countries 2·0 million, uncertainty range 1·8–2·5 million preterm births) compared with 8·6% (total preterm births 2·2 million, 2·0–2·6 million preterm births) in 2010 (table 5)."*<br><br>To avoid this ambiguity, we have removed that sentence from the introduction.
2. I think that it's important for the authors to clarify why they focused on preterm birth – rather than, for example, small for gestational age/low birthweight which, one could argue, is more inclusive & circumvents some of the methodological challenges with calculating preterm births, and is likely relevant to the empowerment variables discussed here.	We belong to an initiative focused on preterm birth and we were asked to write a manuscript on preterm birth, thus the decision to focus on preterm birth was not based on assessment of the most inclusive outcomes . We however think the timing for this paper is right given the increasing attention on prematurity with a number of global initiatives around it. We mention the growing interest in prematurity in the conclusion. The introduction also makes a case for why it is important to consider prematurity. We have added how the discussion may be applicable to other birth outcomes including low birth weight and stillbirths in the last paragraph of the discussion before the limitations
3. In the paragraph beginning "In the preconception period family planning is a highly cost-effective intervention" – note that family planning is, in itself, not an intervention. I would suggest rephrasing this.	We have changed the word "intervention" to "approach."
4. I feel that the first paragraph within "Is there a role of women's empowerment" (beginning "Little is known about how women's empowerment is associated with prematurity") is a result of this analysis, not background information – and should be placed in that section accordingly	We have removed this sentence to maintain consistency in the intent of the paragraph.
5. The first paragraph within Results says that "The only relevant articles from a search on empowerment and prematurity concern research on interventions to 'empower' parents of premature babies in developed settings" – but the second example given (beginning "Recent work in developing settings on group antenatal care (ANC)…") sounds highly relevant so I suggest a rephrase, or else a clearer description of why this is not a valuable example.	We have rephrased both this sentence and the entire section to highlight the available literature.
-Other revisions	
1. I feel it would be helpful for the authors to somewhere mention stillbirths, as these are closely linked to preterm.	We have made mention to stillbirths and other birth outcomes in the last paragraph of the discussion before the limitations
2. In the "Recommended interventions to address the burden of prematurity" section, I am unclear about why	The italics were meant to highlight to steps within the continuum of care: "Preconception period," "antenatal

certain phrases are italicized. If this is meant to refer to steps along the continuum of care, I would suggest doing this consistently (i.e., what's the relationship between "preconception period," "antenatal care," and "skilled attendance"?).	care," and "skilled attendance" for care before pregnancy, during pregnancy and at delivery. As this appears confusing, we have removed the italics.
	We appreciate all your comments and believe they have greatly improved the paper. Thank you for your time and energy in reviewing our work.
**Reviewer #2**	
Overall, I found the paper "Conceptualizing pathways linking women's empowerment and prematurity in developing countries" an interesting and thought-provoking contribution. The authors clearly state the overall aim of the paper, grapple with the dimensions of women's empowerment, and provide important understanding of the known causes and recommended interventions for preterm birth. The review of the relationship between the different dimensions of empowerment and preterm birth and related outcomes could be presented with greater clarity. I provide a number of recommendations toward strengthening the clarity of the presentation and its arguments below toward further strengthening this contribution to the field of maternal and child health.	Thank you for your comments. We have tried to address them as best as we can and respond to them below.<br>We have edited and reorganized the review of the literature and the whole paper to flow better and to be clearer.
-Major Compulsory Revisions	
1. Organization of the paper – I found the arguments at times very hard to follow; and felt that explanation for approaches the authors chose to make came too late, and left too much room for me to question their methods, approaches or decisions. The paper could be significantly strengthened by re-organizing the presentation of some of the sections and where and how the authors introduce different points. There are multiple examples of this. Most importantly:	We have reorganized the entire manuscript to make it easier to follow. See below for the specifics.
a. The presentation of the methods section should precede the discussion on empowerment. We need to understand how you came to learn about empowerment and how it relates to preterm birth. The reader will trust you more if you have already taken us through the steps of your review before you present your conceptualization of empowerment	We have moved the methods section to precede the discussion on empowerment and also expanded on the methods per your recommendation.
b. I would recommend an introduction which includes a review of definition and epidemiology of preterm birth, quickly describes how this differs in high income versus low income settings and then clearly focuses the reader to the low income setting; follow this with methods and then lay out the conceptualization of empowerment for the purposes of understanding	We have made explicit the definition of preterm birth in the introduction. The introduction also includes the epidemiology of preterm birth and how this differs in low and high-income countries and then we present the summary of the risk factors and interventions for preterm birth. The risk factors and interventions for preterm birth apply to both high and high and low

preterm birth.	income countries, but we have highlighted where some are more of concerns in low or high-income countries. , We then follow this with methods and then the conceptualization of empowerment.
c. Presentation of the intervening factors/outcomes that you link to empowerment. Perhaps spend more time on introducing the reader specifically to first pregnancy, spacing, nutrition and stress up front, before you then conceptualize how these link to empowerment. This way the reader will already be convinced of their role in prematurity/preterm birth and then can focus on how empowering women across different dimensions can then improve these outcomes and in turn impact preterm birth. It would also help to include in the methods section how you came to focus on these topics and how you searched for articles on these topics, specifically	We have expanded the discussion on first pregnancy, spacing, nutrition and stress to introduce the reader to these factors up front in the manuscript.<br><br>We also elaborated on our choice of these factors, their importance in developing setting contexts, and why we selected them. We also include in the methods and in the section we present these factors, how and why we selected these factors, and how the search was done for these.
d. Presentation of the figures. It would strengthen the paper if the figures were presented ahead of their being described. Figure 1 could be introduced ahead of your presenting the results for each of these outcomes. This would orient the reader. Through each section you can flesh out how different dimensions and sub-domains of empowerment lead to improved intervening outcomes and then on to impact preterm birth	We have moved presentation of the figures to precede our descriptions of them, and we provided elaboration on how different dimensions of empowerment may improve the intervening outcomes.
e. The presentation of Figure 2. Presenting Figure 1 upfront would then allow you to present Figure 2 when you actually describe the hypothesized linkages between different dimensions of empowerment and stress. For this- State up front that while there is little literature connecting different indicators or measures of empowerment to stress, that findings connecting stress to preterm birth are very strong, and there are plausible links between different dimensions of empowerment and stress, therefore, you will emphasize these links and propose a set of linkages that can then be empirically tested. Make sure this is clear up front – don't want for the discussion section to tell us why you focused on it. This will keep the reader with you.	We have reorganized the section as you have suggested, including presenting Figure 1 upfront and presenting Figure 2 when we describe the hypothesized linkages between different dimensions of empowerment and stress.
2. Conceptualizing Empowerment:	
a. The use of Kabeer is great; it's well-used and well-respected. One place where I see some of your application of Kabeer's work shift from how I have interpreted it, at least, is that you seem to consider empowerment as a *factor*. Kabeer describes it as a *process* that can be conceptualized across several domains each containing sub-domains. There are factors that facilitate empowerment processes. My biggest concern along these lines makes up my next set of comments	Thanks for your suggestions. We have addressed them below.

b. Socio-cultural Empowerment – I simply disagree with how you have framed socio-cultural empowerment. (first paragraph, page 8) For me, many of the aspects that you have captured under socio-cultural empowerment are enablers or facilitators of empowerment, but not dimensions of empowerment themselves. Specifically, gender norms, marriage systems – these are hugely important contextual determinants that when changed can transform a context into one where women can become empowered (or not). Perhaps indicating that these sub-domains fall within 'resources,' or as Kabeer describes resources, the "pre-conditions" to becoming empowered, would help with this. I also think, though, that because the outcomes you are addressing in this paper are very much at an individual level, then there needs to be some recognition of distinctions between community-level indicators versus individual ones. This becomes an issue in your figures where the distinctions between more macro and micro-level processes are not very clear. I just find this 'dimension' would serve better conceptually as a set of contextual factors (as you have indicated in your diagram – what does 'social context' constitute if not these very issues that you are defining as 'socio-cultural empowerment?')	Our framing of these dimensions are based on the works cited. We agree that sociocultural empowerment falls under resources as described by Kabeer and we have added this in the last paragraph of this section.<br><br>Furthermore, all the dimensions can be operationalized at both the individual and community level, although the measures of sociocultural empowerment tend to be more at community level (Malhotra, Schuler, and Boender 2002). Given these are issues of measurement; we are not able to discuss them in this manuscript. We have added a sentence in the last paragraph of this section to highlight that dimensions can be operationalized at individual and community levels and that we consider factors operationalized at the community level as contextual factors.
c. It would also help if you could provide a rationale for the specific dimensions of empowerment you choose to focus on (economic, sociocultural, psychological, and cognitive). Why these, specifically? Have they been explored by others and in this way? (I know the answer for most of these is emphatically yes, but this just needs to be explained, that said, I am not familiar with 'cognitive empowerment,' especially not as it might differ significantly from 'psychological' empowerment.)	Yes, these are dimensions we borrow from prior work and we have added citations and why we chose to use them.<br><br>Cognitive empowerment is used by Stromquist (1995), whom we cite, but as we mention, we also think it is very similar to psychological empowerment, hence the reason we use them together.
d. Perhaps if you draw on Kabeer more closely you could describe socio-cultural empowerment and cognitive empowerment as 'resources' at the community and individual levels, respectively? Somehow the more collective versus individual level dimensions need to be differentiated in the text and ideally in the figures, if possible	From our understanding of the dimensions, socio-cultural and economic empowerment are more in line with resources and psychological and cognitive are more in line with agency. We have added this in the last paragraph of this section.<br><br>As we mention above, all the dimensions can be operationalized at an individual verses collective level, but this is an issue of measurement that can be addressed in empirical applications. We have described this in the last paragraph of this section
e. Distinguishing Autonomy from Empowerment - Autonomy is often used as an indicator for empowerment, especially in the South Asian context where it's understood that it is often denied to women, but be careful not to equate autonomy with	Thank you for your observation. We understand these conceptual distinctions, and we defined autonomy where we make reference to it (reproductive autonomy in second paragraph of section on empowerment and first pregnancy and interpregnancy intervals). However, we

empowerment, or use them interchangeably. It might help to define it as well, which I don't think you do.	included autonomy in our search because in many studies autonomy is often used as an indicator of empowerment.
3. Methods - I find the current description of the methods section inadequate. What databases did you search? How many articles did you find, approximately, that fit the topic? What key words did you use to identify articles on preterm birth? How did you select these? How did you organize the literature that you found? How did you settle on the intervening factors you describe at the top of page 12 in the results section, as those that would then be the focus for this paper?	As noted earlier, this was not intended to be a systematic review. Our initial scoping review did not find relevant articles, particularly for developing settings to warrant a systematic review, which would require that we document all these steps. However, we have provided more details on the steps we did take. We have expanded on the methods to include the databases, key words, and other search criteria for the search on empowerment and prematurity.<br><br>We describe the number of retrieved and reviewed in the results section with how they are organized in the first paragraph of the results section. This section highlights what we found when we searched for empowerment and prematurity.<br><br>We have also added how and why we focused on the intervening factors. As we note, the intervening factors were selected based on our assessment of which linkages were plausible, we drew a lot on previous reviews as well as our prior knowledge of these topic areas. It will be unrealistic to try to do a systematic review for empowerment and all potential intervening factors and so did not try to do that. We have clarified these in the methods and in the section on the intervening factors.
a. Methods in abstract – the language you have in the abstract for methods is not 'methods' to me. It's more a description of your findings. Once you expand the 'how' in the body of the text please use that to guide what you include for the Methods section in the abstract.	We have edited the methods section in the abstract to better fit the intent of the section.
4. Findings – Throughout this section I would encourage you to include more specific language linking specific pathways.	We have tried to do be more specific in how we describe the pathways. Thank you for your recommendation.
a. Stress – as one example, for stress, is there evidence for any of the proposed linkages you cite between different domains of women's empowerment and stress that you can lay out more specifically? For example – economic empowerment: how does employment, or land ownership or escaping poverty, relate to stress? Are their studies that look at different measures of self-efficacy, confidence, decision-making and stress?	We do not have specific evidence for land ownership or escaping poverty and stress, although there are some on poverty in general and stress in developed settings, which we cite. There is some evidence for self-efficacy and stress, although these are also in developed settings. We have cited some of these for our discussion on psychological empowerment and stress. We have added citations where we have supporting evidence. Overall, literature on the links between women's empowerment and stress is lacking both in high and low income countries and we hope this conceptual paper will

	stimulate inquiry and research in this area. We have also expanded the title of this section to psychological health to be more inclusive, as the gap is not just with stress, but with psychological health in general.
b. Services: In this section, I became concerned with what seemed like an assumption that an empowered woman would choose to use health services (last lines of first paragraph). I believe Kabeer cautions about this– I think you have to be a little careful in assuming that an "empowered woman" is going to make the choices that you as healthcare researcher/advocate/provider would like her to make. An empowered mother may choose to marry her daughter before the age of 18; an empowered woman may autonomously choose to deliver her baby at home; exercising agency/self-efficacy. This might help explain how some studies don't find expected linkages between decision-making power, or other forms of autonomy and health service use (as you state on page17, second paragraph).	We state that women's empowerment helps them to overcome the barriers to accessing care ("Women's empowerment helps to mitigate the effects of some of these barriers"); not that it necessarily causes them to choose to use health services, and we cite the literature on the associations between various measures of women's empowerment and use of health services. We also mention the contextual effects of empowerment in the last sentence in this paragraph.
c. On specific language and cognitive empowerment – While knowledge is power, it would strengthen your argument when you describe cognitive empowerment if you were to delineate how the different sets of knowledge you code as cognitive empowerment will then improve a woman's ability to stay healthy and have a healthy baby. In other words- how would these specific bodies of knowledge get transformed into action (which would be more indicative of being empowering)?	It is difficult to refer to specific bodies of knowledge in all references to cognitive empowerment, but where we are able to, we added more specific language.<br><br>For example, we note that "Cognitive empowerment on the other hand may equip women with the knowledge to select healthy foods for themselves and their households" and also that "cognitive empowerment increases perceptions of need for care".
d. Quality of Services- I am not sure this section should be included. There is such a large body of evidence around the relationship between wealth / education and health. We know that wealthier people are healthier and can afford better health services. I think this could be a sentence or two rather than an entire section as the pathway is well worn. Perhaps leave this to the discussion section?	We agree that there is a large body of evidence around the relationship between wealth / education and health, but there isn't that much evidence on empowerment (using the measures of empowerment excluding education and wealth) and quality of care. We used the studies on education and wealth to describe the plausible links, but have taken it out, as it seems not to be helping our point. We have also combined this section with the previous so it is not a section by itself.
-Minor Essential Revisions	
1. Throughout, make sure you cite your sources. You provide examples of psychological empowerment right out of Malhotra et al, but don't cite it there. You have quotes with no citations following them. Check this throughout.	We have added the Malhotra citation and tried to fix this throughout. In most cases where the citation applies to more than one sentence, we put the citation at the end of the last sentence where it applies to avoid being repetitive. Most quotes have a citation at the end of the sentence except when used for emphasis (where we list the key words for example).
2. The opening paragraph of the introduction (page 3) is a bit confusing as it is currently laid out for me, and this may be because I have spent time considering the	The statement on the contribution of fertility is not to preterm birth rates, but to the absolute number of preterm births. That is, high fertility does not increase

issue of fertility decline, specifically. I understand that the intention is to demonstrate that preterm birth is a problem and it's becoming an increasingly serious problem. However, the authors suggest first that preterm birth is more problematic in LMICS than HICs, and suggest that this is probably due to high fertility. Then, the authors suggest that trend data suggest that preterm birth is rising, and cite Latin America as an example- but Latin America has experienced massive, rapid fertility decline, which would suggest that if fertility levels explain higher preterm birth then one would expect the rate of preterm births to be falling, not rising. I think this opening paragraph needs to be stronger, and perhaps should not begin with efforts to explain causality, but simply state what we know about preterm birth rates/trends.	preterm rate but rather the high absolute number of preterm births because there are more births in those settings. Since this statement appears to be confusing, we have taken it out.
3. Fix the use of the word "dimensions" with respect to empowerment on line 129 (third paragraph, page 7) – as you describe other sets of dimensions again on lines 13-138 (first paragraph, page 8). Maybe "components" for line 129?	We used dimensions for both of the areas you highlighted initially because resources, agency, and achievements have been described as dimensions (Kabeer 1999), as have the differences between economic, sociocultural, psychological, and cognitive empowerment. But we understand this may be confusing in the same manuscript and so we have changed the first mention of "dimensions" to "components" as you suggest.
4. Emphasis on Life Course – I would just move this to the Discussion and not suggest up front in the introduction that you will use lifecourse as a specific lens through which you review this literature. It's mentioned far too infrequently for that. The lines between 431 and 438 (second paragraph page 19) seem to come out of nowhere.	We have removed life course from the introduction and due to restructuring of the manuscript, the paragraph mentioned is now in the discussion.
5. Use of the word 'proxy'. I am a bit struck by your use of the word proxy for different measures of empowerment. As a 'proxy' is a 'in lieu of what we'd like to use, we use this' notion, it suggests that we are not able to measure empowerment. As you indicate, there have been MANY efforts to build indices and scales and novel methodologies to try to capture empowerment. I think in many cases you could just describe different 'measures' that capture different dimensions of empowerment, rather than calling them all 'proxies'. In my mind, the more direct measures get at women's control and decision-making, negotiation and address whether and how these have changed over time. The less direct, more appropriately described as 'proxy' measures, are those that measure 'acheivements' alone without an understanding of a woman's role in getting to those achievements; or a sense of how/whether they will be able to benefit her	We are not the first to use proxies for the different measures of empowerment. There have been many efforts to build indices and scales and novel methodologies to try to capture empowerment as empowerment is an abstract construct, and our best efforts are still a representation of what we want to measure. But since its use gives the impression that we have no way of measuring empowerment we have removed it and exchanged the term "proxies" for "measures".

life and ability to make choices to improve it. So, education, income, labor force participation; these to me are more 'proxy' measures than say decision-making, control over resources etc. Mostly, though, I find the repeated use of the word proxy suggests that we have no way of actually capturing empowerment	
a. Lines 132-135 (1st paragraph, page 8): some of the measures you cite as proxy measures here are, in my mind, some of the more direct measures we have (e.g. negotiation/decision-making [if these were change measures])	We have taken out the term "proxy" from the manuscript per this and the above comment.
b. Lines 354-355 (3rd paragraph, page 16) – maybe just "measures"	We have changed the term to measures as suggested.
c. Line 360 (3rd paragraph, page 16) "education and wealth" are not the BEST proxies, they are among the most often used, because they are among the most often measured.	We have taken out the term "best" and used "most often used" per your recommendation.
6. Use of term "empowerment" throughout the paper:- You have done a nice job of showing the reader how complicated and multifaceted 'empowerment' is in the lead up to the presentation of the results; but then don't always maintain this complexity in the presentation of the findings. Please just continue to qualify "empowerment" with phrases like "dimensions of empowerment" or "different measures of empowerment" to maintain this important nuance/complexity	Thank you for this observation. We have made sure to describe empowerment as multi-dimensional and nuanced throughout the manuscript.
a. For example, line 216 – "women who are empowered" … You go on to describe the different dimensions of empowerment, specifically, but it might be stronger if you started with that level of specificity. (e.g. - There is evidence pointing to the importance of the socio-cultural and economic dimensions of women's empowerment in determining outcomes for timing of first birth and interbirth intervals)	We have changed this line to better highlight the dimensions of empowerment from the onset.
b. Another example – line 254 "since empowerment is associated with… " … "it is plausible that empowerment will decrease…" Again, it would be more powerful if you were more specific about which dimensions you had reviewed, or indicated	We have changed this line as well.
7. Findings - Connecting sentences from one section to the next: (e.g. line 258-259) I don't think you need to bring the reader to the next heading like this, I would just finish the topic and then use a new heading to introduce the next. You have already highlighted above that there will be four sets of findings.	This sentence is not just for the sake of connecting the sentences. It is a way of highlighting that the intervening factors are also related and that they may have more indirect effects through other effects. We have edited these, so where it makes sense we reference it in the discussion of the risk factors and removed from the discussion on their relation to empowerment.
8. I admittedly know very little about the topic of preterm birth, but the role of technology and access to advanced medical equipment that comes with wealthy	Technology and access to advanced medical equipment does contribute to the differences between developed and developing countries in the survival of preterm

country contexts seems incredibly important with respect to the differences in mortality levels from preterm births in LMICs as compared to HICs. If this is the case, I think it should be mentioned. This seemed to be a particularly obvious omission on page 4 paragraph 2 where the focus is on the importance of at least a TBA in reducing morbidity/mortality risk associated with prematurity. Perhaps I am too focused on the extreme case (births at 25 weeks); but it would help a lay reader if the authors could explain what the majority-case preterm birth looks like and then how TBAs rather than fully equipped NICU's, can change the outcomes of these births.	babies. But it is unclear where to bring in this discussion, as we are not emphasizing these differences. The focus on empowerment is to shift attention from the clinical to a more social focus. The point of emphasizing receipt of care is that in developing settings many women do not go to deliver in the health facility, and so will not benefit from the advanced technology even if it were there. However we also emphasize good quality care, which will include benefiting from technology where it is available.<br><br>We think going into whether or not the required technology is available and the types of care they could benefit from is beyond the scope of this paper. We think women's empowerment has a role in facilitating access to what is available in all settings.
a. Related, as you are focused on the developing country context, make sure your examples reflect that reality. I wasn't sure on Table 1 if among the causes it was necessary to highlight "increased rates of twins and higher order pregnancies with assisted reproduction." Keep the reader focused on the context you have in mind	"Increased rates of twins and higher order pregnancies with assisted reproduction," is not the most important cause of prematurity in developing countries, but it is still a cause of preterm birth among a minority of women in these settings. Since we are not grouping the causes by prevalence, all the risk factors identified are also risk factors that have been identified for developing countries. We have tried to highlight where they may be less applicable to developing settings.
9. Fix the sentence on lines 38-39 (last sentence paragraph 1, page 4). As stated, it doesn't make sense	The sentence has been changed to make it more understandable.
-Additional Revisions	
1. Discussion - Quality of services - that the quality of care necessary to reduce mortality among preterm birth babies is, I suspect (I don't know the literature) lacking in the developing country setting seems like something that should be addressed more thoroughly; and makes me wonder if prevention is therefore even more important if that would help save more lives if and until better quality of care of is available to the majority of the population in many lower income country settings	Quality of care is indeed important and why we mention it. But even in places where the care is available, few women are accessing the service. This is why we emphasis both use and the quality of the service. Three of the factors in our framework are on prevention and receipt of quality care is the only one that emphasizes both prevention and management of preterm births, which includes care of the preterm baby. Since we cannot prevent all preterm births, it is still useful to discuss care for preterm babies, some of which is just basic essential new born care that is available in developed settings but may not be accessible to many women in the developing setting.
2. One thought I had for a future research project on this topic would be a country-level analysis of the trends between measures of gender equality and rates of pre-term birth, over time	We agree that a country-level analysis will be useful research, and we have added it in the discussion.
I'd like to thank the authors for their hard work. I really enjoyed reading this paper. It pushed my thinking and taught me a lot. Thanks so much.	Thank you.
	We appreciate all your comments and believe they have greatly improved the paper.

Reviewer reports – 2nd round

Reviewer 2: Corrina Moucheraud

- Major Compulsory Revisions
None

- Minor Essential Revisions
I appreciate the extensive work done by the authors to strengthen this manuscript. Thank you very much for taking such time & care in your revisions. I feel the manuscript is vastly improved. I have a few suggestions for changes that should be made before publication; I don't feel that a re-review is necessary, but rather that the article should be accepted pending these revisions.

1. Shouldn't the "conceptualizing women's empowerment" section be in the Introduction? It is not a method per se.
2. Similarly, is the section about "potential intervening factors for a relationship between women's empowerment and prematurity" appropriate in Results? I feel that it should be in the Intro, as an underlying conceptual building block that informed your research approach. The results (in terms of what you found in the lit about each factor etc) belongs in Results -- but, the presentation of the framework itself would be more appropriate in the Intro.
3. I am not sure why the Born Too Soon report (& its infrequent mentions of empowerment) shows up in both Intro & Results. Seems like it should be one or the other.
4. I am unsure whether the "recommended interventions to address the burden of prematurity" section is truly necessary? Although it is obviously important, I felt that it takes the reader away from the main narrative of this paper.
5. I can appreciate the desire to present a parsimonious and elegant diagram -- but I also question the utility of an only semi-complete framework. Perhaps the authors can discuss the need for additional research that fully fleshes out each of these pathways, including connections between intervening factors, etc rather than just stating that you didn't include all possible arrows?

Reviewer 2: Kirsten Stoebenau

Overall the authors have done a terrific job with the edits to this paper. The writing is much clearer (though there are a number of typos that remain, so a solid edit is in order), the overall flow of the paper much easier to follow and the description of the empowerment → intervening factors → prematurity outcomes much easier to read.
That said I do still have a few remaining issues that I think, if resolved, would make the paper quite strong.

Major recommended changes

1. Introduction: You all have done a terrific job of reconfiguring the introduction and set up to the paper. That said, I continue to think that the arguments you make about empowerment and prematurity would be stronger if you kept this paper focused on the prevention of premature labor, and kept the reader from veering toward thoughts about high tech hospital equipment. Your emphasis now on all the causes of prematurity really sets your paper up nicely and I can see how you will be able to build arguments around pathways between empowerment and prematurity via the intervening factors you chose to examine from this set up. But I feel taken "off-track" a bit by the discussion that follows concerning labor/delivery and then even more by the review of interventions once there has been a pre-mature birth (though I do appreciate the caveats you have now added about the availability of high quality services in the developing country setting). Consider eliminating some of this from the introduction as it really doesn't speak to your conceptual framework/broader narrative going forward.

2. Methods: You write:
    a. "Next we examined the literature on the risk factors for prematurity and the recommended interventions to prevent prematurity or improve the survival of premature babies (summarized above); and identified factors from this review that have a plausible link with empowerment, thus could be potential intervening factors between women's empowerment and prematurity. This process was guided by our prior knowledge as well as a scoping review of literature on the determinants of these factors" … but you do not spell out what these factors are. Please do include the list of those factors that you searched as intervening factors.
    b. "We used the same key words for empowerment and added various terms for the intervening factors." Maybe give us an example, this would improve the trustworthiness of this section. For example, for maternal nutrition, we included the following search terms: " …".
3. Empowerment section – I still think the section on empowerment could be a bit stronger. Particularly around how you detail the following: "In addition, all the dimensions can be operationalized at the individual/household, and community level

[19]. In individual level analysis of factors associated with prematurity, many of the measures of empowerment will be at the individual or household level. The community level factors are however very important for analysis at any level and we capture these under contextual factors in our framework." While I appreciate that Kabeer and others suggest that different dimensions of empowerment can manifest at many different levels; you describe economic, psychological and cognitive empowerment at the individual level. The only way one could imagine operationalizing these at another level would be to aggregate individual level data. However, you describe socio-cultural empowerment at the community or macro-level. Gender norms, marriage laws, these are not the property of individuals, but of a collective. That's fine. Can you just please recognize that distinction? It is less obvious (though not impossible) to see how to measure socio-cultural empowerment at the individual level; and likewise, not all that immediately obvious how to measure psychological empowerment at the community level etc.

Minor changes

1. In the abstract, for the methods section:
    a. You should still keep the first sentence which says you conducted a literature review.
    b. You have not yet mentioned intervening factors so this needs a bit of explanation (but I like that you include it here). Maybe you can list out the intervening factors in the paragraph above it?
2. In the introduction, line 174-175 (tracked version) add 'not' to nutritional reserves
3. In the Results – you have done a nice job of explaining why you chose the intervening factors you chose. I wouldn't go too far with "apologizing" for your choices, however. I don't think you need to include why these choices might be biased, for example.
4. Line 599 (tracked version), why the "While"?
5. Make sure you refer readers to the various pathways pointed in Figure 1 as you explain them.
6. I would switch around this sentence on page 29 (tracked version) : "Sociocultural empowerment on the other hand will decrease prematurity by decreasing domestic violence." I would instead write "A decrease in domestic violence, which may result from increased sociocultural empowerment, would decrease prematurity." (in other words, we can't assume that sociocultural empowerment = decreased DV in every case…)
7. There is repetition now on the bottom of page 29 (tracked) you have these two sentences twice within the same paragraph: "In particular, economic empowerment may reduce or eliminate financial strain as a stressor [74,108]. Psychological empowerment on the other hand may be a source of resilience, decreasing the negative effects of stress on birth outcomes [70,109]". In addition, I'm not sure I understand

what aspects of psychological empowerment would be a source of resilience. Can you spell this out more clearly in the paragraphs that follow?

8. Can you spell out a little more what you mean by "women's empowerment" in the following (page 31 tracked version): "This may be a confounding effect of the underlying factors like women's empowerment, but may also suggest an effect of the violence/stress pathways on health care-seeking behaviors." You have just provided such detail on which aspects of women's empowerment would potentially play which roles in reducing stress that this statement at the end of the section sounds like its making too much of a generalization.

Response to reviewers – 2nd round

**Title of manuscript**: "Conceptualizing pathways linking women's empowerment and prematurity in developing countries"	
**Reviewer comment**	**Response**
**Review #2**	
- Minor Essential Revisions<br>I appreciate the extensive work done by the authors to strengthen this manuscript. Thank you very much for taking such time & care in your revisions. I feel the manuscript is vastly improved. I have a few suggestions for changes that should be made before publication; I don't feel that a re-review is necessary, but rather that the article should be accepted pending these revisions.	Thank you. We have tried to address the suggested changes and respond them below
1. Shouldn't the "conceptualizing women's empowerment" section be in the Introduction? It is not a method per se.	We have gone back and forth as to where to put this section given as you rightly state it is not a method. Reviewer 2 however felt strongly about the discussion of the methods preceding the discussion on conceptualizing empowerment, hence we moved it to that section in the first revision. Since this seems to be a matter of preference and is a minor concern for you we think it is safe to leave it in that section in order not to have reorganize in a way that might lead to major concerns in the flow of the paper.
2. Similarly, is the section about "potential intervening factors for a relationship between women's empowerment and prematurity" appropriate in Results? I feel that it should be in the Intro, as an underlying conceptual building block that informed your research approach. The results (in terms of what you found in the lit about each factor etc) belongs in Results -- but, the presentation of the framework itself would be more appropriate in the Intro.	It is difficult to decide the absolute best place for some of the sections since this is not an empirical paper and both the introduction and results are based on a review of the literature. We do however agree that having this section in the intro sets up the paper better and so have moved it to the introduction.
3. I am not sure why the Born Too Soon report (& its infrequent mentions of empowerment) shows up in both Intro & Results. Seems like it should be one or the other.	We have taken out the infrequent mentions of empowerment in the born too soon report from the results
4. I am unsure whether the "recommended interventions to address the burden of prematurity" section is truly necessary? Although it is obviously important, I felt that it takes the reader away from the main narrative of this paper.	Since we mention that we examined recommended interventions, and our framework addresses some of the recommended interventions, it will seem to come from nowhere if we do not mention it in the intro. We have however taken out

	most of the section on interventions after delivery, which might be the part that takes the reader away from the narrative
5. I can appreciate the desire to present a parsimonious and elegant diagram -- but I also question the utility of an only semi-complete framework. Perhaps the authors can discuss the need for additional research that fully fleshes out each of these pathways, including connections between intervening factors, etc rather than just stating that you didn't include all possible arrows?	We did discuss the need for more research to flesh out each of the pathways, and have added a sentence in lines 760-761 to make this more explicit.
	Thank you for all your insightful comments. The paper is much improved because of them.
**Review #2**	
Overall the authors have done a terrific job with the edits to this paper. The writing is much clearer (though there are a number of typos that remain, so a solid edit is in order), the overall flow of the paper much easier to follow and the description of the empowerment → intervening factors → prematurity outcomes much easier to read. That said I do still have a few remaining issues that I think, if resolved, would make the paper quite strong.<br><br>Major recommended changes	Thank you.<br>We have done a thorough edit to remove typos
1. Introduction: You all have done a terrific job of reconfiguring the introduction and set up to the paper. That said, I continue to think that the arguments you make about empowerment and prematurity would be stronger if you kept this paper focused on the prevention of premature labor, and kept the reader from veering toward thoughts about high tech hospital equipment. Your emphasis now on all the causes of prematurity really sets your paper up nicely and I can see how you will be able to build arguments around pathways between empowerment and prematurity via the intervening factors you chose to examine from this set up. But I feel taken "off-track" a bit by the discussion that follows concerning labor/delivery and then even more by the review of interventions once there has been a pre-mature birth (though I do appreciate the caveats you have now added about the availability of high quality services in the developing country setting). Consider eliminating some of this from	We have taken out the discussion of interventions after delivery from the introduction

the introduction as it really doesn't speak to your conceptual framework/broader narrative going forward.	
1. Methods: You write:<br>a. "Next we examined the literature on the risk factors for prematurity and the recommended interventions to prevent prematurity or improve the survival of premature babies (summarized above); and identified factors from this review that have a plausible link with empowerment, thus could be potential intervening factors between women's empowerment and prematurity. This process was guided by our prior knowledge as well as a scoping review of literature on the determinants of these factors" … but you do not spell out what these factors are. Please do include the list of those factors that you searched as intervening factors.<br>2.	We have clarified that we are referring to the determinants of the risk factors and interventions, which we discussed in the introduction
a. "We used the same key words for empowerment and added various terms for the intervening factors." Maybe give us an example; this would improve the trustworthiness of this section. For example, for maternal nutrition, we included the following search terms: " …".	We have added, such as "age at first pregnancy," "interpregnancy interval" "maternal nutrition," "stress," "antenatal care", "skilled birth attendants," etc.
3. Empowerment section – I still think the section on empowerment could be a bit stronger. Particularly around how you detail the following: "In addition, all the dimensions can be operationalized at the individual/household, and community level [19]. In individual level analysis of factors associated with prematurity, many of the measures of empowerment will be at the individual or household level. The community level factors are however very important for analysis at any level and we capture these under contextual factors in our framework." While I appreciate that Kabeer and others suggest that different dimensions of empowerment can	We have expanded this section to highlight the points you make

manifest at many different levels; you describe economic, psychological and cognitive empowerment at the individual level. The only way one could imagine operationalizing these at another level would be to aggregate individual level data. However, you describe socio-cultural empowerment at the community or macro-level. Gender norms, marriage laws, these are not the property of individuals, but of a collective. That's fine. Can you just please recognize that distinction? It is less obvious (though not impossible) to see how to measure socio-cultural empowerment at the individual level; and likewise, not all that immediately obvious how to measure psychological empowerment at the community level etc.	
Minor changes	
1. In the abstract, for the methods section:<br>a. You should still keep the first sentence which says you conducted a literature review.	We have added it, but had to delete the later part of the sentence to stay within the word limit for the abstract
b. You have not yet mentioned intervening factors so this needs a bit of explanation (but I like that you include it here). Maybe you can list out the intervening factors in the paragraph above it?	We have added what we mean by intervening factors. We are unable to expand on it because we exceed the word limit for the abstract when we do so.
2. In the introduction, line 174-175 (tracked version) add 'not' to nutritional reserves	We have added it. Thank you
3. In the Results – you have done a nice job of explaining why you chose the intervening factors you chose. I wouldn't go too far with "apologizing" for your choices, however. I don't think you need to include why these choices might be biased, for example.	We have deleted that sentence
4. Line 599 (tracked version), why the "While"?	Corrected
5. Make sure you refer readers to the various pathways pointed in Figure 1 as you explain them.	It is unclear how to do this without being very repetitive as there are no labels for the various parts of the figure. We have however relabeled the headings in the diagram and the discussion of the literature to be consistent. We also mention that "the

	relationships we posit are shown in figure 1; and we summarize the literature supporting these relationships..” And we refer to the figure where appropriate.
6. I would switch around this sentence on page 29 (tracked version) : “Sociocultural empowerment on the other hand will decrease prematurity by decreasing domestic violence.” I would instead write “A decrease in domestic violence, which may result from increased sociocultural empowerment, would decrease prematurity.” (in other words, we can’t assume that sociocultural empowerment = decreased DV in every case…)	We have changed this.
7. There is repetition now on the bottom of page 29 (tracked) you have these two sentences twice within the same paragraph: “In particular, economic empowerment may reduce or eliminate financial strain as a stressor [74,108]. Psychological empowerment on the other hand may be a source of resilience, decreasing the negative effects of stress on birth outcomes [70,109]”. In addition, I’m not sure I understand what aspects of psychological empowerment would be a source of resilience. Can you spell this out more clearly in the paragraphs that follow?	Repetition removed and we have elaborated on how psychological empowerment can increase resilience
8. Can you spell out a little more what you mean by “women’s empowerment” in the following (page 31 tracked version): “This may be a confounding effect of the <u>underlying factors like women’s empowerment,</u> but may also suggest an effect of the violence/stress pathways on health care-seeking behaviors.” You have just provided such detail on which aspects of women’s empowerment would potentially play which roles in reducing stress that this statement at the end of the section sounds like its making too much of a generalization.	This should say disempowerment as women who experience domestic violence may also be disempowered on other domains. We have clarified this.
	Thank you for your comments. They have improved the paper
